# Clinical and economic burden of nonalcoholic steatohepatitis in Saudi Arabia, United Arab Emirates and Kuwait

**DOI:** 10.1007/s12072-021-10182-x

**Published:** 2021-04-06

**Authors:** Faisal M. Sanai, Abdullah Al Khathlan, Ahmad Al Fadhli, Ahmad S. Jazzar, Al Moutaz Hashim, Eid Mansour, Faisal Abaalkhail, Fuad Hasan, Hajer Al Mudaiheem, Huda Al Quraishi, Juliana Bottomley, Khalid A. Alswat, Mohammed Al Ghamdi, Mohamed Farghaly, Motaz Fathy, Nancy Awad, Omneya Mohamed, Sam Kozma, Waleed Al-Hamoudi, Ahmed Al-jedai

**Affiliations:** 1 Gastroenterology Unit, Department of Medicine, King Abdulaziz Medical City, Jeddah, Saudi Arabia; 2 King Fahd Medical City, Riyadh, Saudi Arabia; 3 Mubarak Al Kabeer Hospital, Jabriya, Kuwait; 4 Sheikh Khalifa Medical City, Abu Dhabi, United Arab Emirates; 5 Department of Medicine, University of Jeddah, Jeddah, Saudi Arabia; 6 Gilead Sciences, Dubai, United Arab Emirates; 7 King Fahad Specialist Hospital, Dammam, Saudi Arabia; 8 Therapeutic Affairs Deputyship, Ministry of Health, Riyadh, Saudi Arabia; 9 Rashid Hospital, Dubai, United Arab Emirates; 10 Gilead Sciences, Stockley Park, Uxbridge, UB11 1AF UK; 11 Department of Medicine, Liver Disease Research Center, College of Medicine, King Saud University, Riyadh, Saudi Arabia; 12 King Fahad Military Medical Complex, Dhahran, Saudi Arabia; 13 Dubai Health Authority, Dubai, United Arab Emirates; 14 IQVIA, Dubai, United Arab Emirates

**Keywords:** Cost, Disease burden, Model, Middle East, NASH, Humanistic, Healthcare expenditure, Patient numbers, Outcomes, Standard of care

## Abstract

**Background and aims:**

The Middle East (ME) has a high prevalence of nonalcoholic fatty liver disease (NAFLD) and nonalcoholic steatohepatitis (NASH), driven by obesity and type-2 diabetes mellitus (T2DM). Studies in Saudi Arabia (KSA) and United Arab Emirates (UAE) predict an escalating impact of NAFLD/NASH, particularly advanced fibrosis due to NASH (AF-NASH), increasing cases of cirrhosis, liver cancer and death. The scale of this burden in other ME countries is unknown with no reports of NAFLD/NASH healthcare resource utilization (HCRU) or costs. We estimated the clinical and economic burden of NAFLD/NASH in KSA, UAE and Kuwait.

**Methods:**

Markov models populated with country-specific obesity and T2DM prevalence data estimated numbers and progression of NAFLD/NASH patients from 2018 to 2030. Model inputs, assumptions and outputs were collected from literature, national statistics, and expert consensus.

**Results:**

Over 13 years, the KSA model estimated cases increasing as follows: patients with fibrosis F0–3 doubled to 2.5 m, compensated and decompensated cirrhosis and hepatocellular carcinoma trebled to 212,000; liver failure or transplant patients increased four-fold to 4,086 and liver-related death escalated from < 10,000 to > 200,000. Similar trends occurred in UAE and Kuwait. Discounted lifetime costs of NASH standard-care increased totaling USD40.41 bn, 1.59 bn and 6.36 bn in KSA, UAE (Emiratis only) and Kuwait, respectively. NASH-related costs in 2019 comprised, respectively, 5.83%, 5.80% and 7.66% of national healthcare spending.

**Conclusions:**

NASH, especially AF-NASH, should be considered a higher priority in ME Public Health policy. Our analyses should inform health policy makers to mitigate the enormity of this escalating regional burden.

**Supplementary Information:**

The online version contains supplementary material available at 10.1007/s12072-021-10182-x.

## Introduction

Chronic liver diseases are an escalating worldwide public-health problem. Of increasing global concern NAFLD, and especially NASH [1, 2], is a leading cause of cirrhosis and advanced liver disease [3, 4]. The most important risk factors driving increasing levels of NAFLD include obesity, T2DM and metabolic syndrome [2, 5]. The consequences of their prevalence mean that NAFLD is the most common chronic liver disease world-wide [6] and a leading cause of liver transplantation (LT) [7].

NAFLD is divided into two sub-types: nonalcoholic fatty liver with a global prevalence of approximately 24% [8] and NASH which is estimated to affect 3–5% of the global population [9]. NASH represents the most progressive form and is characterized by steatosis, inflammation and ballooning, with the accumulation of hepatic fibrosis eventually progressing to decompensated liver disease and/or hepatocellular carcinoma (HCC) in some patients [5, 10]. Liver biopsy is needed to make a definitive diagnosis of NASH [11]. In practice, this invasive procedure is limited in its use due to sampling errors, potential complications and high cost [12], posing challenges for optimal screening and accurate diagnosis of cases. In the absence of well-validated non-invasive tests for NASH [11], diagnosis rates vary considerably across regions [8, 13]. NASH is becoming one of the most common causes of HCC [7, 14], and is the second leading reason for LT in the USA [15]. Recent studies have quantified the enormity of the clinical and economic burden of NAFLD/NASH, with associated HCRU and costs, particularly in AF-NASH, predicting these costs will grow [16–20].

Pandemic levels of obesity [21] and T2DM [22] in the KSA, UAE and Kuwait (with average body mass indices of 28.5, 28.8 and 30.0, respectively) [21] together with their aging populations suggest that NAFLD/NASH-associated advanced liver disease and mortality will increase in these countries [23, 24]. NAFLD prevalence in the ME is 32% [8], one of the highest worldwide, although there are limited data for individual countries. Available reports suggest at least a third of the Kuwaiti, UAE and KSA population suffer from NAFLD (33.3% [19], 34.7% [25] and ≤ 52% [26, 27], respectively).

Alswat et al. [23] assessed the clinical burden of NAFLD/NASH in KSA and UAE and predicted significant increases in cases with advanced liver disease and mortality attributable to NASH by 2030; however, the associated clinical burden in Kuwait has not been assessed in detail. Recent Kuwaiti data showed 63% of patients referred for LT in 2018–2019 had NASH-related cirrhosis [28].

It is essential to have a clearer understanding of the impact of this disease in the ME. Considering the paucity of evidence, a simulation model was developed to understand the current and future burden of illness of NASH. This would allow informed decision-making on allocating resources for developing optimal interventions for NASH treatment. Furthermore, clarity on the disease burden may also drive the providers, payers, and policy makers to design NASH identification algorithms, especially for those at high-risk of disease progression.

## Materials and methods

### Model structure

**Fig. 1:**
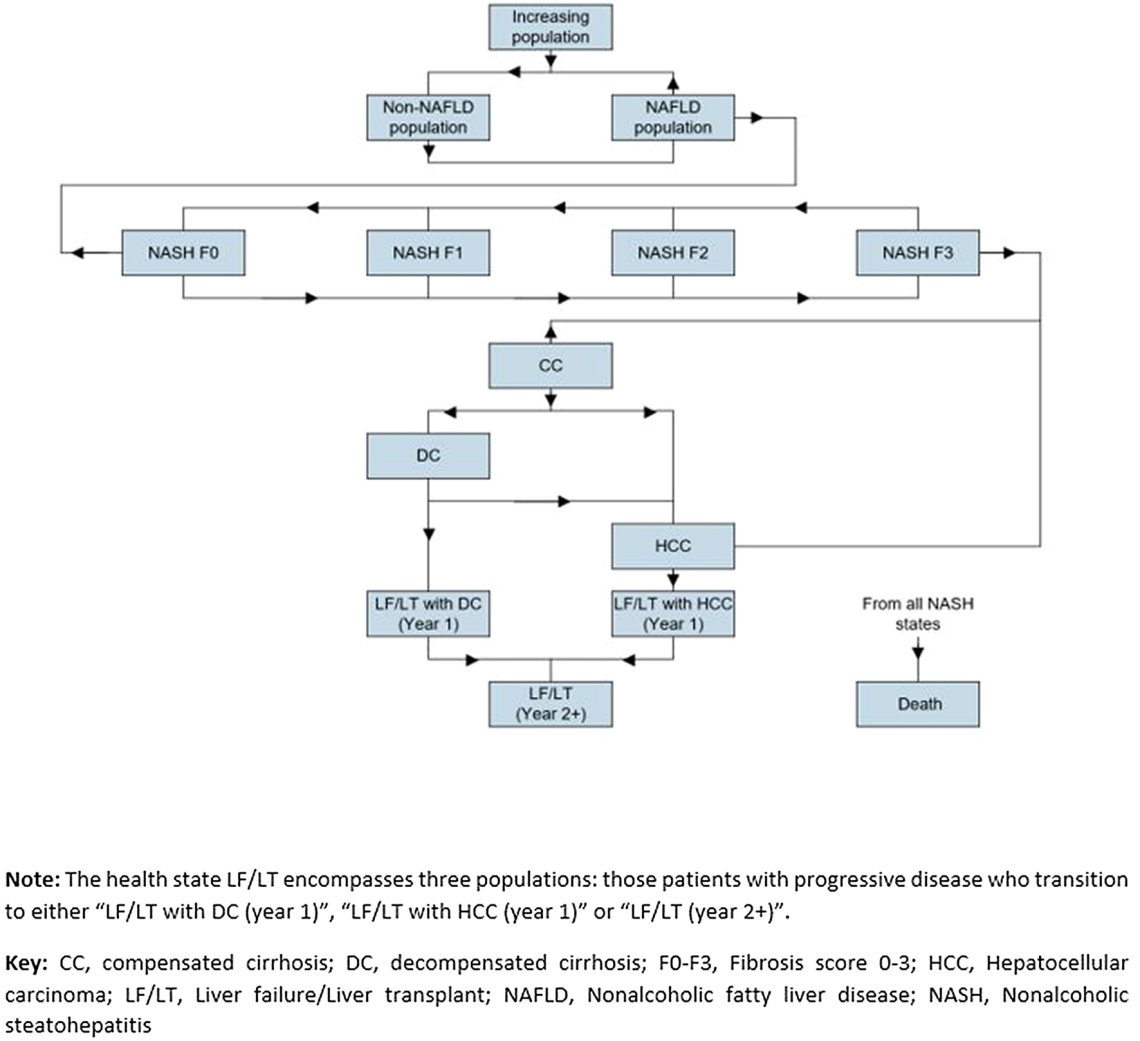
Model structure

The complexity of NAFLD/NASH and patients entering the model over time was simulated using a Markov Model (Fig. 1). The total number of patients increased annually based on population projections for the KSA and UAE23, and from United Nations projections for Kuwait [29]. At baseline, patients were separated into those with or without NAFLD (who may have developed NASH). Prior to the development of cirrhosis, NASH patients were categorized by fibrosis score (ranging from F0 to F3). F3 patients may progress to cirrhosis (F4). Compensated cirrhosis (CC) may progress to decompensated cirrhosis (DC) or HCC; however, some patients may develop HCC directly from NASH F0 to F3. DC patients may develop HCC or require LT. After developing DC, patients were at increased risk of liver-related death if not offered LT. The health-state LF/LT encompassed three populations; patients with progressive disease who transitioned to health-states, “LF/LT with DC (year 1)”, “LF/LT with HCC (year 1)” or “LF/LT (year 2 +)” simulating liver failure and subsequent liver transplant due to DC “fail DC” and liver failure and subsequent liver transplant due to HCC “fail HCC”. In year 1, these were separated into two health states (LF/LT due to DC and LF/LT due to HCC) as patients with HCC have a higher probability of liver failure than the patients with DC. In subsequent years, these patients were combined into a “liver failure year 2 + ” health state. All patients were at risk of non-liver related death throughout the model. It was assumed that the increasing general population applied to the non-NASH group of patients.

### Model assumptions

Transition probabilities were calculated by re-calibrating the projections reported in Alswat [23] (model calibration steps are seen in Online Resource 1, Supplementary Fig. 1). The same transition probabilities were applied for all three countries. All-cause mortality was assumed to be 1% per year from all health-states [30].

### Time horizon and population

The model considers a period similar to Alswat [23]. In the base case, a starting year of 2018 was applied for a 13-year time horizon. It also considers the same patient populations for KSA and UAE [23]. For KSA, the Alswat model accounted for all patients residing in the country (both nationals and expatriates) but UAE considered Emirati citizens only [23]. The UAE population considered in our model also comprised local citizens only; Emiratis represent 11.5% of the UAE population [31]. Limited data are available regarding the NASH population for Kuwait; therefore, estimates from the World Bank were used. The estimated local population size for the UAE and total population size for Kuwait and KSA in 2017 were 1,020,000, 4,136,528 and 32,900,000, respectively [23, 29].

### Model inputs

Detailed country-specific standard of care (SoC) in terms of drugs used, tests, contacts with healthcare professionals (HCPs), hospital admissions, procedures and LTs per model health-state was elicited from 21 national policy-makers or medical experts responsible for treating NAFLD/NASH patients. Any disagreements were resolved via consensus. HCRU was estimated for 1st year and subsequent years to account for management at diagnosis and that recurring thereafter in KSA, UAE and Kuwait (Online Resource 1, Supplementary Tables 1–6). Corresponding unit costs were sought in local currencies and in USA dollars (USD, 2018) (Online Resource 1, Supplementary Tables 7 and 8). An annual discount rate of 3% was applied to all costs. In the absence of ME-specific utility values, UK utility values [32] informed the health-states (Online Resource 1, Supplementary Table 9).

## Results

### Patients.

**Fig. 2:**
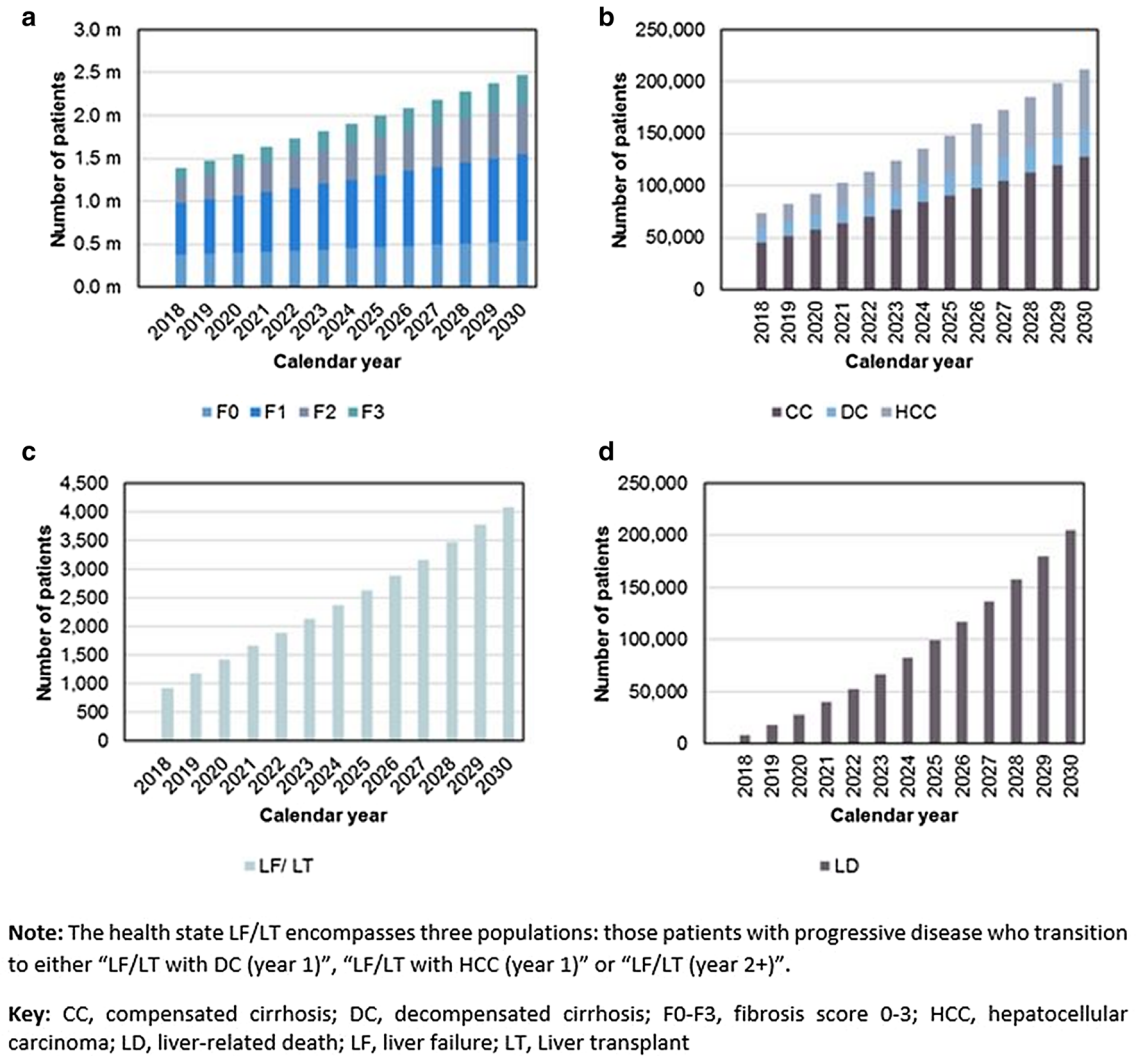
New cases annually per model health-state in KSA. NASH patients on Standard of Care (2018–2030): **a** fibrosis stages F0, F1, F2 and F3; **b** compensated cirrhosis, decompensated cirrhosis and hepatocellular carcinoma; **c** liver failure or liver transplant and **d** liver-related death

Over the simulation period, the increasing number of new cases per health-state on SoC in KSA are shown in Fig. 2. The model estimated patients with F0–F3 increasing annually from 1.39 m in 2018 to 2.47 m in 2030 (78% increase, Fig. 2a), cases with CC, DC and HCC increasing from 73,368 to 212,070 (189% increase, Fig. 2b), LF or LT increasing > fourfold from 930 to 4086 in 2030 (Fig. 2c) with cases of liver-related death escalating from 8227 in 2018 to 204,000 in 2030 (Fig. 2d). Similar trends in escalating new cases were also predicted in UAE and Kuwait (Online Resource 2, Supplementary Figs. 1 and 2, respectively).

### Quality-adjusted life years (QALYs)

Given that the KSA, UAE and Kuwaiti models adopted the same transition probabilities for SoC management, the proportions of QALYs accrued in each health-state were equal in all countries with the majority accrued in initial fibrosis stages of NASH. It is also clear that with disease progression, very few QALYs accrued (Online Resource 2, Supplementary Table 1 and Supplementary Fig. 3, respectively).

### Costs

**Fig. 3:**
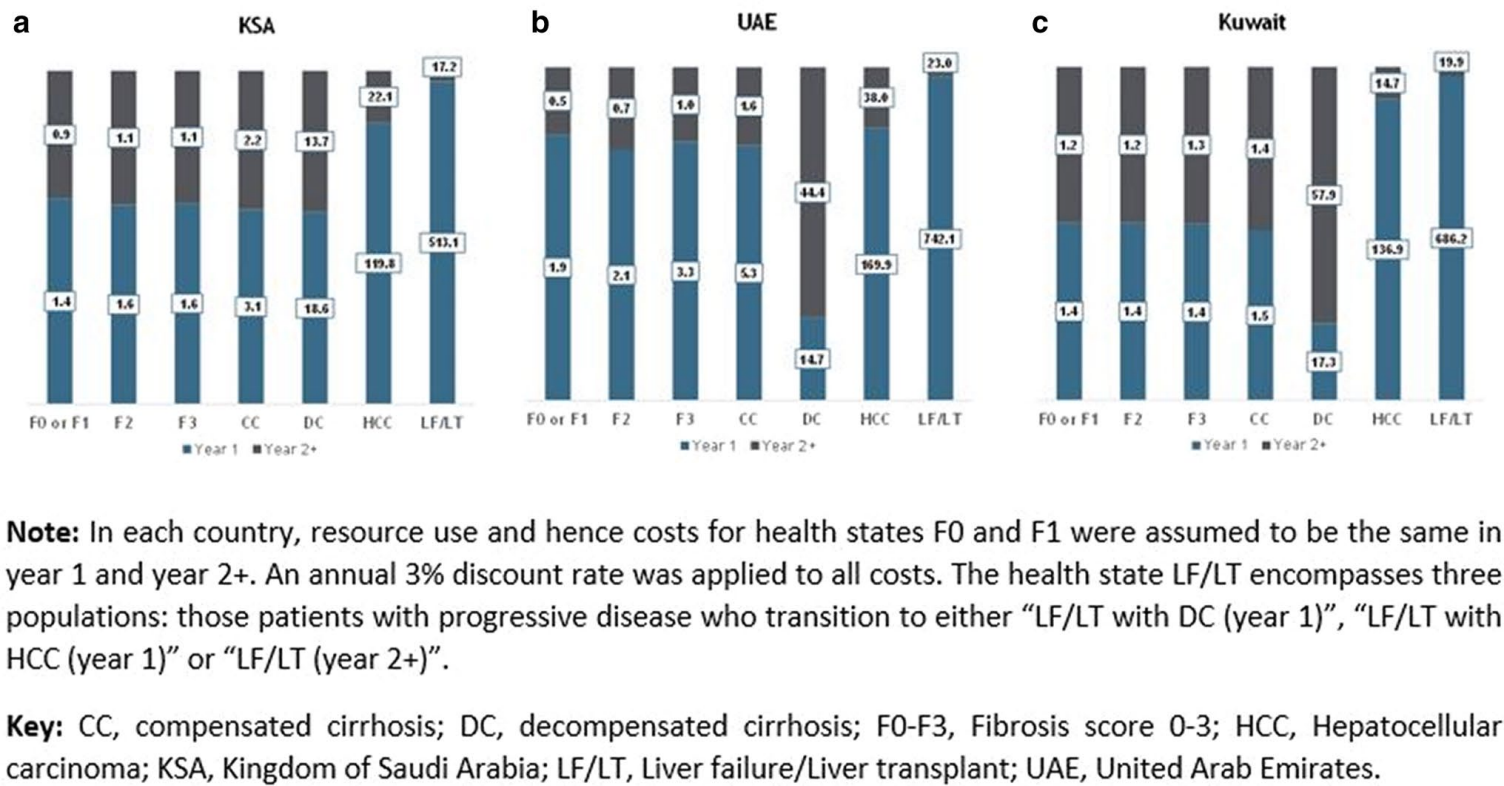
Annual patient health-state model costs. NASH patients on Standard of Care—1st-Year and Subsequent Years: **a** KSA, **b** UAE and **c** Kuwait [USD (thousands)]

Figure 3 presents the annual patient costs of SoC per NASH health-state (for 1st year and subsequent years) in KSA, UAE and Kuwait. Across all three countries, annual costs in year 1 were higher than subsequently. There was an exponential increase in the healthcare costs with disease progression. Figure 3 shows that relative to F1, the total annual costs for the health-states of HCC and LF/LT were significantly higher in all countries per defined population. In KSA (Fig. 3a), costs of a patient in health-state HCC were USD119,773 in year 1 (86-fold greater than F1), and USD22,111 (25-fold larger than F1) subsequently. LF/LT annual per patient costs were USD513,148 in year 1 (371-fold greater than F1), and USD17,244 (20-fold greater than F1) in subsequent years (Country-specific model annual costs per health-state in local currencies are seen in Online Resource 2, Supplementary Table 2).

Table 1 presents total lifetime discounted NASH SoC patient costs per population for KSA, UAE and Kuwait according to (i) annual costs from 2018 to 2030 and (ii) each model health-state. The model predicted lifetime national costs of USD40.41 bn, USD1.59 bn and USD6.36 bn in KSA, UAE and Kuwait, respectively, up to 2030 assuming continuing SoC management (with predicted lifetime costs in local currencies seen in Online Resource 2, Supplementary Table 3).

**Table 1:** Discounted Population Costs (USDmillions). NASH patients on Standard of Care in KSA, UAE and Kuwait (i) Annual costs 2018–2030, (ii) Lifetime costs per health-state—all years

(i) Annual costs 2018–2030														
Country	Year													Total
	2018	2019	2020	2021	2022	2023	2024	2025	2026	2027	2028	2029	2030	
KSA	2351.7	2483.7	2615.9	2747.3	2877.1	3004.7	3129.6	3251.5	3369.8	3484.4	3595.0	3701.2	3803.0	40,414.8
UAE^a^	90.2	95.4	100.7	106.2	111.7	117.3	122.9	128.5	133.9	139.3	144.6	149.7	154.7	1595.2
Kuwait	395.9	408.9	423.3	438.6	454.7	471.2	488.0	504.9	521.8	538.4	554.7	570.7	586.2	6357.5

(ii) Lifetime costs per health-state—all years											
Country	Health-State										Total
	F0	F1	F2	F3	CC	DC	HCC	LF/LT with DC (year 1) Fail DC	LF/LT with HCC (year 1) Fail HCC	LF/LT (year 2 +) Fail Y2 +	
KSA	5020.3	7973.1	5087.0	3196.7	2414.5	3021.4	10,736.7	1065.0	1730.5	169.6	40,414.8
UAE^a^	141.8	174.5	118.1	132.3	99.6	256.5	540.0	47.8	77.6	7.0	1595.2
Kuwait	782.7	1346.1	698.3	423.7	161.4	1347.7	1103.1	179.1	290.8	24.6	6357.5

Health-state LF/LT encompasses three populations: patients with progressive disease transitioning to “LF/LT with DC (year 1)”, “LF/LT with HCC (year 1)” or “LF/LT (year 2 +)”

*CC* compensated cirrhosis, *DC* decompensated cirrhosis, *Fail DC* liver failure with decompensated cirrhosis, *Fail HCC* liver failure with hepatocellular carcinoma, *Fail Y2*+ liver failure for 2 or more years, *F0-F3* fibrosis score 0–3, *HCC* hepatocellular carcinoma, *KSA* Kingdom of Saudi Arabia, *NASH* nonalcoholic steatohepatitis, *UAE* United Arab Emirates

^a^Local Emiratis population only

**Fig. 4:**
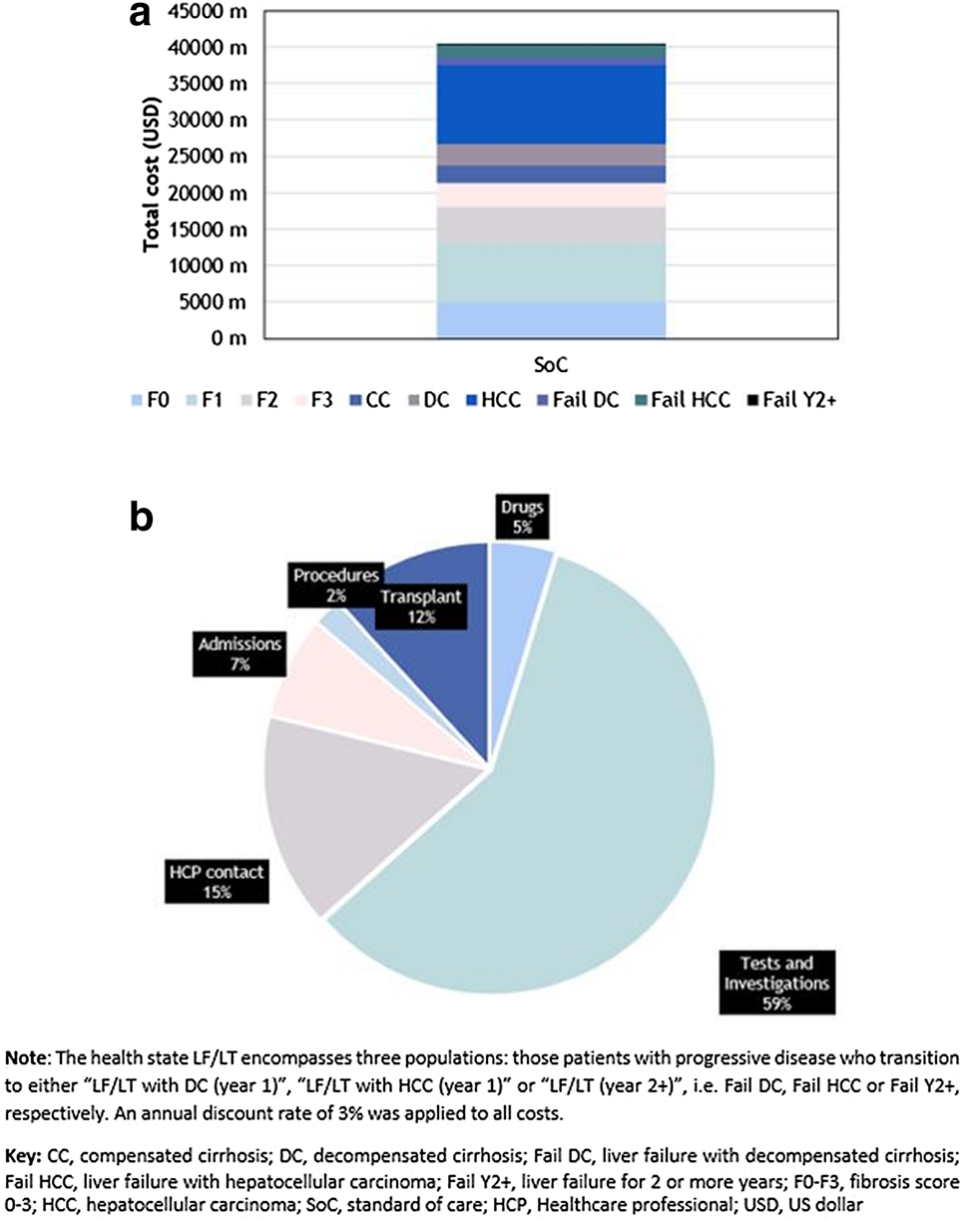
Breakdown of costs (USD). NASH patients on Standard of Care in KSA from 2018 to 2030. **a** Health-state; **b** cost category

In KSA per model health state, Table 1 and Fig. 4a demonstrate that most of the 2018–2030 expenditure is incurred in the HCC (USD10.7 bn) and F1 (USD7.97 bn) health-states. Although per patient annual costs with F1 are small relative to advanced fibrosis (Fig. 3), the lifetime costs and burden of F1 at a population level are high due to far larger number of F1 cases; there were an estimated 1 million patients with NASH F1 and 56,700 cases with HCC in KSA in 2030. Across all NASH health-states from F0 through to LF/LT (i.e., patients with fail DC, fail HCC or fail Y2 +), in terms of HCRU across the KSA NASH population, most of the lifetime costs (Fig. 4b) are due to tests and investigations (59%), HCP care time (15%) and LT (12%). However, in the earlier stages of NASH (F0-CC), the main driver of cost is tests and investigations, whereas for the more advanced stages of AF-NASH or HCC, the in-hospital HCRU (linked to admissions, procedures and transplants) become more prominent. Analogous results in UAE and Kuwait based on local currency model simulations are seen in Online Resource 2, Supplementary Figs. 5 and 6, respectively. Similar breakdown of cost estimates was predicted in Kuwait although in UAE the proportion of costs for tests and investigations was estimated to be less (Online Resource 2, Supplementary Fig. 5b).

In all countries, the annual discounted spend for NASH patients treated with SoC increased over time. In 2019 the estimated annual costs of managing the NASH population was USD2483.7 m, USD95.4 m and USD408.9 m increasing to USD3803.0 m, USD154.7 m and USD586.2 m in KSA, UAE and Kuwait in 2030, respectively (Table 1).

## Discussion

This study provides a comprehensive estimate of the clinical and economic burden of NASH and AF-NASH, if expert-informed SoC management remains unchanged, in adult ME populations. It shows that NASH and particularly the complications of cirrhosis, HCC and liver-related death present a serious ongoing pressure on healthcare expenditures. Our models for KSA, UAE and Kuwait predict alarming surges in the NASH caseloads in the coming decade, which warrant immediate attention and urgent action.

The lifetime discounted direct costs associated with NASH and AF-NASH managed with SoC increased dramatically across all countries, amounting to USD40.4 bn, USD1.6 bn and USD6.36 bn in KSA, UAE (local population) and Kuwait, respectively, through 2030, reflecting the increasing country caseloads over the period. These lifetime cost estimates equate to an overall national health spend for NASH of USD1230 in KSA, USD1570 in UAE (local population) and USD1530 in Kuwait per head of population, as defined in our populations analysis. Our models predict significantly increased number of NASH patients in KSA, UAE and Kuwait with earlier F0–F3 compared with the more advanced fibrosis stages, and this is driving the mounting caseload per country. Burgeoning patient numbers explain the significant proportion of cost incurred by patients with stages F0–F1 nationally. The larger proportion of health spend for the HCC patients is driven by higher individual per patient costs compared to earlier stages of disease, this being driven by higher in-hospital costs.

Younossi et al. [18] also estimated the total economic burden of NASH and advanced NASH in the USA using a lifetime economic model. They predicted incident and prevalent lifetime cost for all NASH cases in 2017 to be USD222.6 bn with the costs of the advanced NASH population being USD95.4 bn [18]. Their overall total direct NASH costs equate to around USD890 health spend per head of population (based upon the USA adult population of 249.49 million) [18]. The authors concluded NASH was associated with high lifetime economic burden and in the absence of treatment these direct costs will continue to grow [18].

In our analyses, annual costs associated with management of SoC NASH cases per country increased year on year. When compared to national healthcare budgets for 2019 (USD42.56 bn in KSA [33], USD14.31 bn in UAE [34] (and adjusted health spend in Emirati citizens of USD1.64 bn) and USD5.34bn in Kuwait [35]) our 2019 analyses point to the potential of NASH consuming 5.83% and 5.80% as a proportion of health spend in KSA and UAE and even more in Kuwait (7.66%). Based upon budget forecasts in 2024 the trend is for this proportion to increase in KSA to 5.9% (Online Resource 3, Supplementary Table 1), assuming NASH patient management remains unchanged.

Younossi et al. [36] in 2016 estimated annual direct medical costs of some USD103bn in over 64 million people projected to have NAFLD in USA and about USD35bn in Germany, France, Italy and UK with some 52 million people with NAFLD. Their estimates for NASH cases were USD16.75bn in USA and USD6.16bn in Europe-4 [36]. By referencing their country estimates for total annual direct costs of NASH in 2016 and relevant national statistics of health spend and national populations, we calculate their analyses reflect a 2016 national percentage of health spend due to NASH of 0.55%, 0.19%, 0.68%, 1.08% and 0.36% in USA, Germany, France, Italy and UK, respectively (Online Resource 3, Supplementary Table 2).

Our study suggests that each ME country will need to allocate over 5% of its entire annual health spend to combat the wave of NASH and its sequelae. Most of the costs incurred for NASH SoC management are for the advanced stages of the disease, namely DC and HCC, which while representing less than 2% of the NASH population contribute to some 50% of the costs. The escalating caseload of F0–F3 cases also contributes significantly to the spiraling projected costs. With the numbers of earlier fibrosis stages as well as HCC cases projected to increase significantly across all three countries through 2030 based on no change in patient management, the need for urgent attention and action is clear.

It is well established that the trajectory of NASH parallels that of increasing obesity and T2DM prevalence, and ME countries have some of the highest prevalence of adult obesity and T2DM world-wide; this may contribute to the higher percentage ME health spend predicted in our modelling compared to US and European studies.

The impact on quality of life is of significance in NASH [37]. This detrimental effect was clearly demonstrated in our modelling via the sobering QALY loss with progression from early fibrosis stages to AF-NASH.

Recognition of the need for action is seen in transformational health policy initiatives across the region [38–40]. ME countries are regarded as high-income developing countries; Kuwait was ranked 9^th^ in the global Gross Domestic Product per capita list [41]. It is also recognized by governments that the abundance of financial resources can increase the likelihood of over-nutrition and hinder a more active work-life balance—both risk factors for NASH and for Public Health initiatives aimed at their prevention.

Currently there are no pharmacologic treatments for NASH that go beyond non-specific supportive care. Should an effective treatment become available, the potential health gain and the national cost savings because of delaying and/or preventing progression to the costly poorer prognosis states may be substantial.

Our analyses under-estimate the overall economic impact of NASH and its complications; other NASH patient comorbidities and associated HCRU and costs, non-medical costs and wider societal costs were not included in the modelling. Our study is subject to several other limitations. Model simulations are based upon optimal evidence and assumptions, and both are subject to inadequacies. Our approach was in accord with Alswat et al. [23] and by default its associated limitations. In the absence of any national data or literature, relevant assumptions in the models were informed by extensive country expert input. Nevertheless, our findings resonate with and extend the findings to a wider ME region.

The impact of NASH is a matter of grave concern and must be regarded as such by all national healthcare systems. That the Ministries of Health are putting initiatives forward to control diseases is welcome. This serious liver disease can no longer be perceived as a lifestyle disease. Regional clinical experts recently raised concerns regarding the national statistics of NASH and their impact and have called for urgent government and non-government organization action [24, 28]. Importantly, proposed ME diagnostic and management roadmaps, respectful of cost and local expertise consideration, could contribute toward an action plan to identify and improve the SoC for people with NASH and AF-NASH [28].

Our study based its assumptions on the current situation and a continuation of SoC unchanged. However, the situation is rapidly worsening considering the regional pandemic of obesity and T2DM. There must be an immediate effort and commitment to address the increasing challenges and unmet needs facing the ME medical community. Actions need to be broad-ranging, comprehensive and collaborative with multidisciplinary input ensuring widespread adoption [24, 28]. Areas for consideration to tackle issues of disease management and to raise its priority could include:

1. *Creation and implementation of a national plan for NAFLD* to ensure HCPs, payers and medical professionals are aligned on the optimal screening, diagnostics, management, and monitoring of NAFLD/NASH patients. Plans should capture important activities and outcomes in annual Ministry of Health statistics.
2. *Prevention and educational strategies* to educate the public about NAFLD/NASH as a chronic progressive liver disease, associated outcomes and risk factors and increase visibility of health awareness programs for people to help themselves. Two such plans include the Rashaka [42] program in KSA (which aims to reduce obesity rates 5% by 2020) and the observance of Anti-Obesity day in the UAE [43].
3. *Disease surveillance* to provide policy makers with a detailed demographic “big picture” of the current circumstances, data that can be utilized to validate statistical models and economic analyses and allow better data extrapolations. The identification and screening of high-risk individuals is key: Patients having risk factors that predispose to NAFLD/NASH should be identified and tracked. National and region-wide patient registries of such individuals would have multiple benefits and would contribute to a better understanding of epidemiology, management, and outcomes.
4. *Novel therapies* Access to eventual new innovative treatments could come via support from all regional healthcare stakeholders via encouraging and funding the creation and validation of novel interventions that may present.

We believe our study reinforces the call for higher Public Health priority for NASH, and especially AF-NASH across all ME countries. The authors propose avenues to consider which could slow and ultimately reverse its negative burden, where these steps are immediate and targeted.

## Supplementary Information

Below is the link to the electronic supplementary material.

Supplementary file1 (DOCX 1175 kb)

Supplementary file2 (DOCX 520 kb)

Supplementary file3 (DOCX 48 kb)

## Data Availability

The online version of this article contains supplementary material supporting the results. Any additional data is available from the corresponding author upon request.

## Abbreviations

AF-NASH: Advanced fibrosis due to NASH
CC: Compensated cirrhosis
DC: Decompensated cirrhosis
F: Fibrosis
HCC: Hepatocellular carcinoma
HCRU: Healthcare resource utilization
HCP: Healthcare professional
KSA: Kingdom of Saudi Arabia
LD: Liver-related death
LF: Liver failure
LT: Liver transplant
LY: Life year
ME: Middle East
NAFLD: Nonalcoholic fatty liver disease
NASH: Nonalcoholic steatohepatitis
SoC: Standard of care
T2DM: Type-2 diabetes mellitus
QALY: Quality adjusted life year
UAE: United Arab Emirates
USD: United States Dollar

## Declarations

### Clinical Trials Registration

Not applicable. This article does not contain any studies with human subjects (or animals).
