# Supplementary material for: Clinical and economic burden of nonalcoholic steatohepatitis in Saudi Arabia, United Arab Emirates and Kuwait
Source: Hepatol Int. 2021 Apr 6;15(4):912–21. doi: 10.1007/s12072-021-10182-x (PMC8382637; doi:10.1007/s12072-021-10182-x)
Supplement: Supplementary file 1 — Supplementary file1 (DOCX 1175 kb) [file 12072_2021_10182_MOESM1_ESM.docx]

**ARTICLE TITLE: Clinical and economic burden of nonalcoholic steatohepatitis in Saudi Arabia, United Arab Emirates and Kuwait**

**JOURNAL NAME**: Hepatology International

**AUTHOR NAMES**: Faisal M Sanai, Abdullah Al Khathlan, Ahmad Al Fadhli, Ahmad S Jazzar, Al Moutaz Hashim, Eid Mansour, Faisal Abaalkhail, Fuad Hasan, Hajer Al Mudaiheem, Huda Al Quraishi, Juliana Bottomley, Khalid A Alswat, Mohammed Al Ghamdi, Mohamed Farghaly, Motaz Fathy, Nancy Awad, Omneya Mohamed,

**CORRESPONDING AUTHOR**: Professor Ahmed Al-jedai, Therapeutic Affairs Deputyship, Ministry of Health, Saudi Arabia. E-mail: [ahaljedai@moh.gov.sa](mailto:ahaljedai@moh.gov.sa)

**SUPPLEMENTARY MATERIAL 1. Model Inputs**

1. Model Calibration Steps
2. Model Inputs Per Health State for NASH Patients on Standard of Care in KSA, UAE and Kuwait
   1. Annual Health Care Resource Use Frequencies
   2. Health Care Costs
   3. Utilities

**1.Model Calibration**

Model calibration was performed using the built-in Solver functionality of Microsoft^®^ Excel. Firstly, the results presented by Alswat *et al.* (i.e. projected patient numbers) were extracted using graph digitization software (GetData Graph Digitizer)^[[1]](#endnote-1)^. After extracting the data, a transition matrix was set up with permittable transitions. The possible transitions were included following consultation with clinical experts in the KSA (who advised that it was possible for patients to progress from NASH F0-F3 to develop HCC). Furthermore, the occurrence of liver failure was separated into two health states dependent on whether the patient previously had DC or HCC (as a higher probability of liver failure is anticipated for HCC patients versus DC patients). In subsequent years, these patients were combined into a “liver failure year 2+” health state.

A mathematical program was then formalized with the objective of minimizing the difference in the modelled and digitized estimates of patient numbers in each health state per year. The program included constraints pertaining to the non-negativity of each plausible transition probability, as well as the requirement of all possible transitions from a given health state to sum to a total of 100% (i.e. that all transition probabilities satisfied the law of total probability).

Finally, the program was executed using the Solver add-in and the results were checked for accuracy by visual assessment. The evolutionary algorithm was utilized within Solver owing to the non-linearity of the model itself (i.e. an increase of one unit for a given probability does not lead to an equivalent increase or decrease in the objective function [in this case, the sum of the absolute error]). This process is summarized in Supplementary Figure 1.

**Supplementary Figure 1 - Model calibration process**


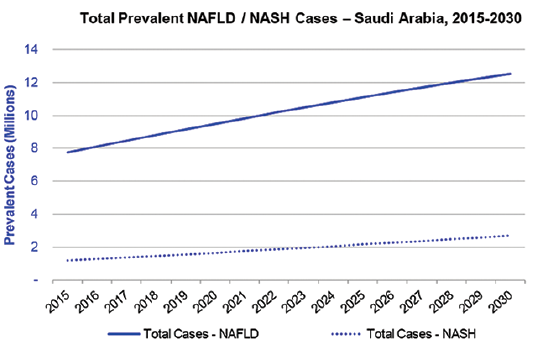

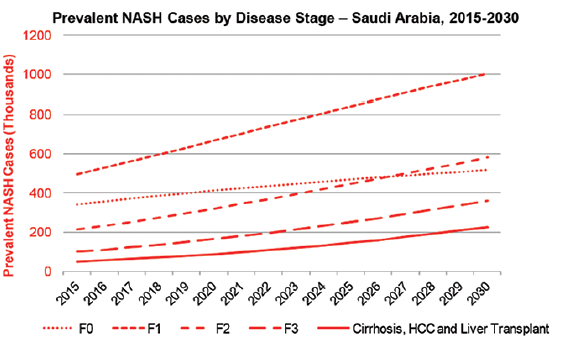


Step 1: Digitise data from Alswat, 2018


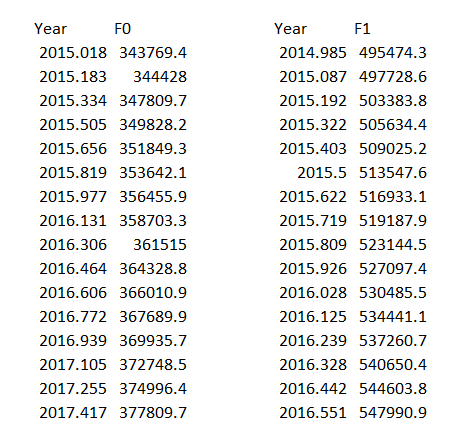


Step 2: Set up transition matrix with permittable transitions


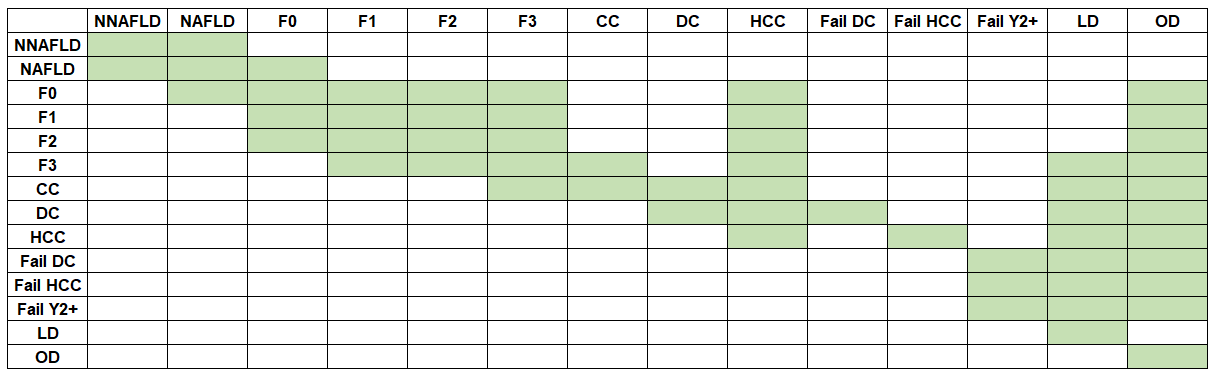


Step 3: Formalise the program

$$Minimise \sum_{State=1}^{14} \sum_{Year=2017}^{2030} \left( {Digitised number}_{Year} -{Modelled number}_{Year} \right)$$

$$Subject to: 0 \leq{Transition}_{i, j} \leq1 \forall i,j \in\left\{ 0, 1, \ldots, 14 \right\}$$

Where i is the state the transition is from, and j is the state the transition is to

Step 4: Run program and check accuracy


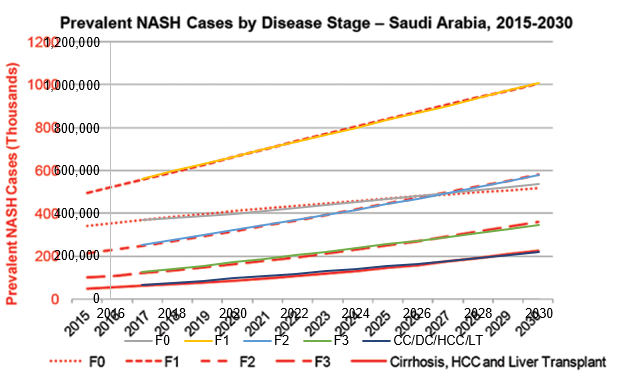

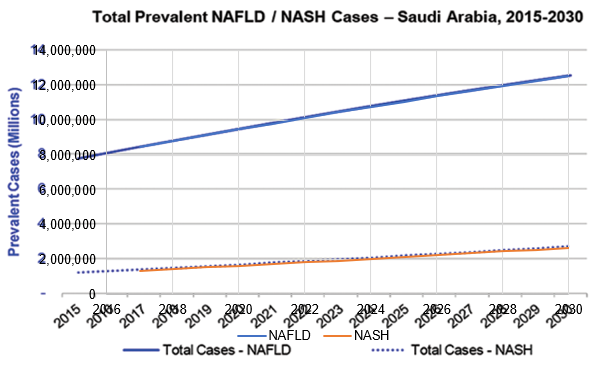


**2.Model Inputs**

**I. Health care resource use (HCRU) Frequencies per health state for NASH patients on Standard of Care in KSA, UAE and Kuwait**

Clinician interviews informed typical patient management HCRU model inputs for NASH patients receiving Standard of Care (SoC) in all three countries.

**KSA**: The expert estimated HCRU for NASH patients receiving SoC in Year 1 (Supplementary Table 1) and in subsequent years (Supplementary Table 2) in KSA in order to account correctly for HCRU associated with SoC in KSA specifically at diagnosis and those that reoccur annually thereafter. The main difference between year 1 and subsequent years in KSA was that aside from fibroscans and liver function tests, other liver tests were not used. In addition, the use of several drugs was stopped (e.g. mycophenolate mofetil, Valgan) or greatly reduced (e.g. tacrolimus, cyclosporin, prednisolone).

**UAE**: Supplementary Table 3 and Supplementary Table 4 describe HCRU inputs for UAE (year 1 and subsequent years, respectively).

**Kuwait**: Supplementary Table 5 and Supplementary Table 6 present the corresponding model inputs for Kuwait.

In summary, very similar HCRU to KSA in year 1 were evident in both UAE and Kuwait. Furthermore, observations of similar subtle test and drug use changes in subsequent years were evident in both UAE and Kuwait.

Supplementary Table 1. Annual HCRU Frequencies Per Health State for NASH patients on SoC in KSA (Year 1)

| HCRU | | Health States | | | | | | | | | | | |
| --- | --- | --- | --- | --- | --- | --- | --- | --- | --- | --- | --- | --- | --- |
| Category | Resource | NNAFLD | NAFLD | F0 | F1 | F2 | F3 | CC | DC | HCC | Fail DC* | Fail HCC* | Fail Y2+* |
| Drug | Vitamin E |  |  | 694 | 694 | 694 | 694 |  |  |  |  |  |  |
|  | Pioglitazone |  |  |  |  |  | 183 | 183 |  |  |  |  |  |
|  | Acamprosate |  |  |  |  |  |  |  |  |  |  |  |  |
|  | Obeticholic Acid |  |  |  |  |  |  |  |  |  |  |  |  |
|  | Carvedilol |  |  |  |  |  |  |  |  |  |  |  |  |
|  | Multivitamins |  |  |  |  |  |  | 365 |  |  |  |  |  |
|  | Thiamine |  |  |  |  |  |  |  | 365 |  |  |  |  |
|  | Spironolactone |  |  |  |  |  |  |  | 365 | 73 |  |  |  |
|  | Furosemide |  |  |  |  |  |  |  | 365 | 73 |  |  |  |
|  | Rifaximin |  |  |  |  |  |  |  | 731 |  |  |  |  |
|  | Lactulose |  |  |  |  |  |  |  | 292 | 58 |  |  |  |
|  | Ceftriaxone |  |  |  |  |  |  |  | 8 |  |  |  |  |
|  | Octreotide |  |  |  |  |  |  |  | 7 |  |  |  |  |
|  | Normal Saline |  |  |  |  |  |  |  | 33 |  |  |  |  |
|  | Terlipressin |  |  |  |  |  |  |  | 3 |  |  |  |  |
|  | IV Albumin |  |  |  |  |  |  |  | 4 |  |  |  |  |
|  | Midodrine |  |  |  |  |  |  |  | 2 |  |  |  |  |
|  | Mycophenolate mofetil |  |  |  |  |  |  |  |  |  | 365 | 365 |  |
|  | Tacrolimus |  |  |  |  |  |  |  |  |  | 2630 | 2630 |  |
|  | Cyclosporine |  |  |  |  |  |  |  |  |  | 7305 | 7305 |  |
|  | Prednisolone |  |  |  |  |  |  |  |  |  | 183 | 183 |  |
|  | Valgan |  |  |  |  |  |  |  |  |  | 30 | 30 |  |
|  | Sorafenib |  |  |  |  |  |  |  |  | 95 |  |  |  |
|  | Nivolumab |  |  |  |  |  |  |  |  | 0.11 |  |  |  |
|  | New Treatment |  |  |  |  |  | 365 | 365 |  |  |  |  |  |
| Tests | Fibroscan |  |  | 0.67 | 0.67 | 2 | 2 | 1 |  |  |  |  |  |
|  | Liver Function Test |  |  | 2 | 2 | 2 | 2 | 2 | 4 | 6 |  |  |  |
|  | Hep B/C serology |  |  | 1 | 1 | 1 | 1 | 1 | 2 |  |  |  |  |
|  | Ultrasound |  |  | 1 | 1 | 1 | 1 | 2 | 2 |  |  |  |  |
|  | Liver Biopsy |  |  |  |  |  |  |  |  | 1 |  |  |  |
|  | Autoimmune Liver Screen |  |  | 1 | 1 | 1 | 1 |  | 3 |  |  |  |  |
|  | OGD |  |  |  |  |  |  | 1 | 5 |  |  |  |  |
|  | Ultrasound for HCC |  |  |  |  |  |  | 2 |  |  |  |  |  |
|  | Alpha Fetoprotein |  |  |  |  |  |  | 2 |  | 3 |  |  |  |
|  | Metabolic markers |  |  | 1 | 1 | 1 | 1 | 1 | 2 |  |  |  |  |
|  | CBC |  |  | 1 | 1 | 1 | 1 | 2 | 4 |  |  |  |  |
|  | Blood chemistry |  |  | 1 | 1 | 1 | 1 | 2 | 4 |  |  |  |  |
|  | Coagulation profile |  |  | 1 | 1 | 1 | 1 | 2 | 4 | 6 |  |  |  |
|  | Creatinine clearance |  |  | 1 | 1 | 1 | 1 | 2 | 4 |  |  |  |  |
|  | HBs AG-6 |  |  |  |  |  |  |  |  | 1 |  |  |  |
|  | HCV antibody-6 |  |  |  |  |  |  |  |  | 1 |  |  |  |
|  | CT/MRI scan |  |  |  |  |  |  |  |  | 3 |  |  |  |
| Contact with HCPs | GP Appointment |  |  | 2 | 2 | 2 | 2 | 2 | 4 |  |  |  |  |
|  | Dietician Appointment |  |  | 1 | 1 | 1 | 1 | 1 | 1 |  |  |  |  |
|  | Consultant Outpatient Visit |  |  |  |  |  | 1 |  |  |  |  |  |  |
|  | Outpatient Visits |  |  |  |  |  |  | 2 |  | 4 |  |  |  |
|  | Nurse Telephone Consultations |  |  |  |  |  |  |  |  | 3 |  |  |  |
|  | Hospital Admission - Follow-up |  |  |  |  |  |  |  |  | 1 |  |  |  |
|  | Day Case Follow-up |  |  |  |  |  |  |  |  | 1 |  |  |  |
|  | Outpatient Visits* |  |  |  |  |  |  |  | 4 |  |  |  |  |
| Admissions | Emergency Admission - Ascites |  |  |  |  |  |  |  | 3 |  |  |  |  |
|  | Emergency Admission - Variceal Bleed |  |  |  |  |  |  |  | 1 |  |  |  |  |
|  | Emergency Admission - Encephalopathy |  |  |  |  |  |  |  | 1 |  |  |  |  |
|  | *Planned Admission - Ascites |  |  |  |  |  |  |  | 0.5 |  |  |  |  |
|  | Planned Admission - Variceal Bleeds |  |  |  |  |  |  |  | 0.5 |  |  |  |  |
|  | Hospital Admission - Tumour recurrence |  |  |  |  |  |  |  |  | 0.5 |  |  |  |
| Procedures | TIPS Stent |  |  |  |  |  |  |  | 0.01 |  |  |  |  |
|  | Day Case, Ascites |  |  |  |  |  |  |  | 6 |  |  |  |  |
|  | Surgical re-section |  |  |  |  |  |  |  |  |  |  | 0.05 |  |
|  | Radiofrequency ablation (RFA) |  |  |  |  |  |  |  |  | 0.03 |  |  |  |
|  | TransArterial chemoembolization (TACE) |  |  |  |  |  |  |  |  | 0.3 |  |  |  |
|  | TransArterial radioembolization (TARE) |  |  |  |  |  |  |  |  | 0.3 |  |  |  |
|  | Paracentesis |  |  |  |  |  |  |  |  | 0.2 |  |  |  |
| Transplant | Liver Transplant |  |  |  |  |  |  |  |  | 0.3 | 1 | 1 |  |
|  | Liver Re-transplantation |  |  |  |  |  |  |  |  |  |  |  |  |
|  | Subsequent Care |  |  |  |  |  |  |  |  |  | 0.95 | 0.95 |  |
| HCC, hepatocellular carcinoma; HCRU, Health Care Resource Use; HCP, Health Care Professional; KSA, Kingdom of Saudi Arabia; OGD, Oesophago-gastroduodenoscopy; SoC, Standard of Care; TIPS, Transjugular intrahepatic portosystemic shunt.  **Note**: *The health state LF/LT encompasses three populations: those patients with progressive disease who transition to either “LF/LT with DC (year 1)”, “LF/LT with HCC (year 1)” or “LF/LT (year 2+)”, i.e. Fail DC, Fail HCC and Fail Y2+, respectively.**For Ascites, Variceal Bleeds or Encephalopathy. | | | | | | | | | | | | | |

Supplementary Table 2. Annual HCRU frequencies per health state for NASH patients on SoC in KSA (subsequent years)

| HCRU | | Health States | | | | | | | | | | | |
| --- | --- | --- | --- | --- | --- | --- | --- | --- | --- | --- | --- | --- | --- |
| Category | Resource | NNAFLD | NAFLD | F0 | F1 | F2 | F3 | CC | DC | HCC | Fail DC* | Fail HCC* | Fail Y2+* |
| Drug | Vitamin E |  |  | 694 | 694 | 694 | 694 |  |  |  |  |  |  |
|  | Pioglitazone |  |  |  |  |  | 183 | 183 |  |  |  |  |  |
|  | Acamprosate |  |  |  |  |  |  |  |  |  |  |  |  |
|  | Obeticholic Acid |  |  |  |  |  |  |  |  |  |  |  |  |
|  | Carvedilol |  |  |  |  |  |  |  |  |  |  |  |  |
|  | Multivitamins |  |  |  |  |  |  | 365 |  |  |  |  |  |
|  | Thiamine |  |  |  |  |  |  |  | 365 |  |  |  |  |
|  | Spironolactone |  |  |  |  |  |  |  | 365 |  |  |  |  |
|  | Furosemide |  |  |  |  |  |  |  | 365 |  |  |  |  |
|  | Rifaximin |  |  |  |  |  |  |  | 731 |  |  |  |  |
|  | Lactulose |  |  |  |  |  |  |  | 292 |  |  |  |  |
|  | Ceftriaxone |  |  |  |  |  |  |  | 8 |  |  |  |  |
|  | Octreotide |  |  |  |  |  |  |  | 7 |  |  |  |  |
|  | Normal Saline |  |  |  |  |  |  |  | 33 |  |  |  |  |
|  | Terlipressin |  |  |  |  |  |  |  | 3 |  |  |  |  |
|  | IV Albumin |  |  |  |  |  |  |  | 4 |  |  |  |  |
|  | Midodrine |  |  |  |  |  |  |  | 2 |  |  |  |  |
|  | Mycophenolate mofetil |  |  |  |  |  |  |  |  |  |  |  |  |
|  | Tacrolimus |  |  |  |  |  |  |  |  |  |  |  | 904 |
|  | Cyclosporine |  |  |  |  |  |  |  |  |  |  |  | 2511 |
|  | Prednisolone |  |  |  |  |  |  |  |  |  |  |  | 114 |
|  | Valgan |  |  |  |  |  |  |  |  |  |  |  |  |
|  | Sorafenib |  |  |  |  |  |  |  |  |  |  |  |  |
|  | Nivolumab |  |  |  |  |  |  |  |  |  |  |  |  |
|  | New Treatment |  |  |  |  |  | 365 | 365 |  |  |  |  |  |
| Tests | Fibroscan |  |  | 0.67 | 0.67 | 2 | 2 | 1 |  |  |  |  |  |
|  | Liver Function Test |  |  | 2 | 2 | 2 | 2 | 2 | 4 | 6 |  |  |  |
|  | Hep B/C serology |  |  |  |  |  |  |  | 2 |  |  |  |  |
|  | Ultrasound |  |  |  |  |  |  | 2 | 2 |  |  |  |  |
|  | Liver Biopsy |  |  |  |  |  |  |  |  |  |  |  |  |
|  | Autoimmune Liver Screen |  |  |  |  |  |  |  | 3 |  |  |  |  |
|  | OGD |  |  |  |  |  |  | 0.54 | 5 |  |  |  |  |
|  | Ultrasound for HCC |  |  |  |  |  |  | 2 |  |  |  |  |  |
|  | Alpha Fetoprotein |  |  |  |  |  |  | 2 |  | 3 |  |  |  |
|  | Metabolic markers |  |  |  |  |  |  |  | 2 |  |  |  |  |
|  | CBC |  |  |  |  |  |  | 2 | 4 |  |  |  |  |
|  | Blood chemistry |  |  |  |  |  |  | 2 | 4 |  |  |  |  |
|  | Coagulation profile |  |  |  |  |  |  | 2 | 4 | 6 |  |  |  |
|  | Creatinine clearance |  |  |  |  |  |  | 2 | 4 |  |  |  |  |
|  | HBs AG-6 |  |  |  |  |  |  |  |  | 1 |  |  |  |
|  | HCV antibody-6 |  |  |  |  |  |  |  |  | 1 |  |  |  |
|  | CT/MRI scan |  |  |  |  |  |  |  |  | 3 |  |  |  |
| Contact with HCPs | GP Appointment |  |  | 2 | 2 | 2 | 2 | 2 | 4 |  |  |  |  |
|  | Dietician Appointment |  |  |  |  |  |  | 1 | 1 |  |  |  |  |
|  | Consultant Outpatient Visit |  |  |  |  |  |  |  |  |  |  |  |  |
|  | Outpatient Visits |  |  |  |  |  |  |  |  | 4 |  |  |  |
|  | Nurse Telephone Consultations |  |  |  |  |  |  |  |  | 3 |  |  |  |
|  | Hospital Admission - Follow-up |  |  |  |  |  |  |  |  |  |  |  |  |
|  | Day Case Follow-up |  |  |  |  |  |  |  |  |  |  |  |  |
|  | Outpatient Visits** |  |  |  |  |  |  |  | 4 |  |  |  |  |
| Admissions | Emergency Admission - Ascites |  |  |  |  |  |  |  | 3 |  |  |  |  |
|  | Emergency Admission - Variceal Bleed |  |  |  |  |  |  |  | 1 |  |  |  |  |
|  | Emergency Admission - Encephalopathy |  |  |  |  |  |  |  | 1 |  |  |  |  |
|  | Planned Admission - Ascites |  |  |  |  |  |  |  | 0.5 |  |  |  |  |
|  | Planned Admission - Variceal Bleeds |  |  |  |  |  |  |  | 0.5 |  |  |  |  |
|  | Hospital Admission - Tumour recurrence |  |  |  |  |  |  |  |  | 0.5 |  |  |  |
| Procedures | TIPS Stent |  |  |  |  |  |  |  | 0.01 |  |  |  |  |
|  | Day Case, Ascites |  |  |  |  |  |  |  |  |  |  |  |  |
|  | Surgical re-section |  |  |  |  |  |  |  |  |  |  |  |  |
|  | Radiofrequency ablation (RFA) |  |  |  |  |  |  |  |  |  |  |  |  |
|  | TransArterial chemoembolization (TACE) |  |  |  |  |  |  |  |  |  |  |  |  |
|  | TransArterial radioembolization (TARE) |  |  |  |  |  |  |  |  |  |  |  |  |
|  | Paracentesis |  |  |  |  |  |  |  |  |  |  |  |  |
| Transplant | Liver Transplant |  |  |  |  |  |  |  |  |  |  |  |  |
|  | Liver Re-transplantation |  |  |  |  |  |  |  |  |  |  |  | 0.05 |
|  | Subsequent Care |  |  |  |  |  |  |  |  |  |  |  | 0.95 |
| HCC, hepatocellular carcinoma; HCP, Health Care Professional; HCRU, Healthcare resource use; KSA, Kingdom of Saudi Arabia; OGD, Oesophago-gastroduodenoscopy; SoC, Standard of Care; TIPS, Transjugular intrahepatic portosystemic shunt;  **Note**: * The health state LF/LT encompasses three populations: those patients with progressive disease who transition to either “LF/LT with DC (year 1)”, “LF/LT with HCC (year 1)” or “LF/LT (year 2+)”, i.e. Fail DC, Fail HCC and Fail Y2+, respectively. **For Ascites, Variceal Bleeds or Encephalopathy. | | | | | | | | | | | | | |

Supplementary Table 3. Annual HCRU frequencies per health state for NASH patients on SoC in UAE (year 1)

| HCRU | | Health States | | | | | | | | | | | |
| --- | --- | --- | --- | --- | --- | --- | --- | --- | --- | --- | --- | --- | --- |
| Category | Resource | NNAFLD | NAFLD | F0 | F1 | F2 | F3 | CC | DC | HCC | Fail DC* | Fail HCC* | Fail Y2+* |
| Drug | Vitamin E |  |  | 694 | 694 | 694 | 694 |  |  |  |  |  |  |
|  | Pioglitazone |  |  |  |  |  | 183 | 183 |  |  |  |  |  |
|  | Acamprosate |  |  |  |  |  |  |  |  |  |  |  |  |
|  | Obeticholic Acid |  |  |  |  |  |  |  |  |  |  |  |  |
|  | Carvedilol |  |  |  |  |  |  |  |  |  |  |  |  |
|  | Multivitamins |  |  |  |  |  |  | 365 |  |  |  |  |  |
|  | Thiamine |  |  |  |  |  |  |  | 365 |  |  |  |  |
|  | Spironolactone |  |  |  |  |  |  |  | 365 | 73 |  |  |  |
|  | Furosemide |  |  |  |  |  |  |  | 365 | 73 |  |  |  |
|  | Rifaximin |  |  |  |  |  |  |  | 731 |  |  |  |  |
|  | Lactulose |  |  |  |  |  |  |  | 292 | 58 |  |  |  |
|  | Ceftriaxone |  |  |  |  |  |  |  | 7.5 |  |  |  |  |
|  | Octreotide |  |  |  |  |  |  |  | 6.8 |  |  |  |  |
|  | Normal Saline |  |  |  |  |  |  |  | 32.6 |  |  |  |  |
|  | Terlipressin |  |  |  |  |  |  |  | 2.63 |  |  |  |  |
|  | IV Albumin |  |  |  |  |  |  |  | 3.5 |  |  |  |  |
|  | Midodrine |  |  |  |  |  |  |  | 1.8 |  |  |  |  |
|  | Mycophenolate mofetil |  |  |  |  |  |  |  |  |  | 365 | 365 |  |
|  | Tacrolimus |  |  |  |  |  |  |  |  |  | 2630 | 2630 |  |
|  | Cyclosporine |  |  |  |  |  |  |  |  |  | 7305 | 7305 |  |
|  | Prednisolone |  |  |  |  |  |  |  |  |  | 183 | 183 |  |
|  | Valgan |  |  |  |  |  |  |  |  |  | 30 | 30 |  |
|  | Sorafenib |  |  |  |  |  |  |  |  | 95 |  |  |  |
|  | Nivolumab |  |  |  |  |  |  |  |  | 0.1 |  |  |  |
|  | New Treatment |  |  |  |  |  | 365 | 365 |  |  |  |  |  |
| Tests | Fibroscan |  |  | 0.67 | 0.67 | 2 | 2 | 1 |  |  |  |  |  |
|  | Liver Function Test |  |  | 2 | 2 | 2 | 2 | 2 | 4 | 6 |  |  |  |
|  | Hep B/C serology |  |  | 1 | 1 | 1 | 1 | 1 | 2 |  |  |  |  |
|  | Ultrasound |  |  | 1 | 1 | 1 | 1 | 2 | 2 |  |  |  |  |
|  | Liver Biopsy |  |  |  |  |  |  |  |  | 1 |  |  |  |
|  | Autoimmune Liver Screen |  |  | 1 | 1 | 1 | 1 | 1 | 4 |  |  |  |  |
|  | OGD |  |  |  |  |  |  | 2 | 2 |  |  |  |  |
|  | Ultrasound for HCC |  |  |  |  |  |  | 2 |  |  |  |  |  |
|  | Alpha Fetoprotein |  |  |  |  |  |  | 2 |  | 3 |  |  |  |
|  | Metabolic markers |  |  | 1 | 1 | 1 | 1 | 1 | 4 |  |  |  |  |
|  | CBC |  |  | 1 | 1 | 1 | 1 | 2 | 4 |  |  |  |  |
|  | Blood chemistry |  |  | 1 | 1 | 1 | 1 | 2 | 4 |  |  |  |  |
|  | Coagulation profile |  |  | 1 | 1 | 1 | 1 | 2 | 4 | 6 |  |  |  |
|  | Creatinine clearance |  |  | 1 | 1 | 1 | 1 | 2 | 4 |  |  |  |  |
|  | HBs AG-6 |  |  |  |  |  |  |  |  | 4 |  |  |  |
|  | HCV antibody-6 |  |  |  |  |  |  |  |  | 1 |  |  |  |
|  | CT/MRI scan |  |  |  |  |  |  |  |  | 2.7 |  |  |  |
| Contact with HCP | GP Appointment |  |  | 2 | 2 | 2 | 2 | 2 | 4 |  |  |  |  |
|  | Dietician Appointment |  |  | 1 | 1 | 1 | 1 | 1 | 1 |  |  |  |  |
|  | Consultant Outpatient Visit |  |  |  |  |  | 1 |  |  | 3 |  |  |  |
|  | Outpatient Visits |  |  |  |  |  |  | 2 |  | 5.5 |  |  |  |
|  | Nurse Telephone Consultations |  |  |  |  |  |  |  |  |  |  |  |  |
|  | Hospital Admission - Follow-up |  |  |  |  |  |  |  |  | 1 |  |  |  |
|  | Day Case Follow-up |  |  |  |  |  |  |  |  | 1 |  |  |  |
|  | Outpatient Visits** |  |  |  |  |  |  |  | 5.5 |  |  |  |  |
| Admissions | Emergency Admission - Ascites |  |  |  |  |  |  |  | 2 |  |  |  |  |
|  | Emergency Admission - Variceal Bleed |  |  |  |  |  |  |  | 1.5 |  |  |  |  |
|  | Emergency Admission - Encephalopathy |  |  |  |  |  |  |  | 2 |  |  |  |  |
|  | Planned Admission - Ascites |  |  |  |  |  |  |  | 0.5 |  |  |  |  |
|  | Planned Admission - Variceal Bleeds |  |  |  |  |  |  |  | 0.5 |  |  |  |  |
|  | Hospital Admission - Tumour recurrence |  |  |  |  |  |  |  |  | 1 |  |  |  |
| Procedures | TIPS Stent |  |  |  |  |  |  |  | 0.02 |  |  |  |  |
|  | Day Case, Ascites |  |  |  |  |  |  |  | 1.2 |  |  |  |  |
|  | Surgical re-section |  |  |  |  |  |  |  |  |  |  | 0.05 |  |
|  | Radiofrequency ablation (RFA) |  |  |  |  |  |  |  |  | 0.02 |  |  |  |
|  | TransArterial chemoembolization (TACE) |  |  |  |  |  |  |  |  | 0.3 |  |  |  |
|  | TransArterial radioembolization (TARE) |  |  |  |  |  |  |  |  | 0.3 |  |  |  |
|  | Paracentesis |  |  |  |  |  |  |  |  | 0.2 |  |  |  |
| Transplant | Liver Transplant |  |  |  |  |  |  |  |  | 0.3 | 1 | 1 |  |
|  | Liver Re-transplantation |  |  |  |  |  |  |  |  |  |  |  |  |
|  | Subsequent Care |  |  |  |  |  |  |  |  |  | 0.95 | 0.95 |  |
| HCC, hepatocellular carcinoma; HCP, Health care professional; OGD, HCRU, Healthcare Resource Use; Oesophago-gastroduodenoscopy; SoC, Standard of Care; TIPS, Transjugular intrahepatic portosystemic shunt; UAE, United Arab Emirates  **Note**: * The health state LF/LT encompasses three populations: those patients with progressive disease who transition to either “LF/LT with DC (year 1)”, “LF/LT with HCC (year 1)” or “LF/LT (year 2+)”, i.e. Fail DC, Fail HCC and Fail Y2+, respectively. **For Ascites, Variceal Bleeds or Encephalopathy. | | | | | | | | | | | | | |

Supplementary Table 4. Annual HCRU frequencies per health state for NASH patients on SoC in UAE (subsequent years)

| HCRU | | Health States | | | | | | | | | | | |
| --- | --- | --- | --- | --- | --- | --- | --- | --- | --- | --- | --- | --- | --- |
| Category | Resource | NNAFLD | NAFLD | F0 | F1 | F2 | F3 | CC | DC | HCC | Fail DC* | Fail HCC* | Fail Y2+* |
| Drug | Vitamin E |  |  | 694 | 694 | 694 | 694 |  |  |  |  |  |  |
|  | Pioglitazone |  |  |  |  |  | 183 | 183 |  |  |  |  |  |
|  | Acamprosate |  |  |  |  |  |  |  |  |  |  |  |  |
|  | Obeticholic Acid |  |  |  |  |  |  |  |  |  |  |  |  |
|  | Carvedilol |  |  |  |  |  |  |  |  |  |  |  |  |
|  | Multivitamins |  |  |  |  |  |  | 365 |  |  |  |  |  |
|  | Thiamine |  |  |  |  |  |  |  | 365 |  |  |  |  |
|  | Spironolactone |  |  |  |  |  |  |  | 365 |  |  |  |  |
|  | Furosemide |  |  |  |  |  |  |  | 365 |  |  |  |  |
|  | Rifaximin |  |  |  |  |  |  |  | 731 |  |  |  |  |
|  | Lactulose |  |  |  |  |  |  |  | 292 |  |  |  |  |
|  | Ceftriaxone |  |  |  |  |  |  |  | 7.5 |  |  |  |  |
|  | Octreotide |  |  |  |  |  |  |  | 6.8 |  |  |  |  |
|  | Normal Saline |  |  |  |  |  |  |  | 32.6 |  |  |  |  |
|  | Terlipressin |  |  |  |  |  |  |  | 2.6 |  |  |  |  |
|  | IV Albumin |  |  |  |  |  |  |  | 3.5 |  |  |  |  |
|  | Midodrine |  |  |  |  |  |  |  | 1.8 |  |  |  |  |
|  | Mycophenolate mofetil |  |  |  |  |  |  |  |  |  |  |  |  |
|  | Tacrolimus |  |  |  |  |  |  |  |  |  |  |  | 328.7 |
|  | Cyclosporine |  |  |  |  |  |  |  |  |  |  |  | 36.5 |
|  | Prednisolone |  |  |  |  |  |  |  |  |  |  |  | 114.1 |
|  | Valgan |  |  |  |  |  |  |  |  |  |  |  |  |
|  | Sorafenib |  |  |  |  |  |  |  |  |  |  |  |  |
|  | Nivolumab |  |  |  |  |  |  |  |  |  |  |  |  |
|  | New Treatment |  |  |  |  |  | 365 | 365 |  |  |  |  |  |
| Tests | Fibroscan |  |  | 0.67 | 0.67 | 2 | 2 | 1 |  |  |  |  |  |
|  | Liver Function Test |  |  |  |  |  |  | 2 | 2 | 6 |  |  |  |
|  | Hep B/C serology |  |  |  |  |  |  |  | 2 |  |  |  |  |
|  | Ultrasound |  |  |  |  |  |  |  | 2 |  |  |  |  |
|  | Liver Biopsy |  |  |  |  |  |  |  |  |  |  |  |  |
|  | Autoimmune Liver Screen |  |  |  |  |  |  |  | 4 |  |  |  |  |
|  | OGD |  |  |  |  |  |  |  | 4 |  |  |  |  |
|  | Ultrasound for HCC |  |  |  |  |  |  | 2 |  |  |  |  |  |
|  | Alpha Fetoprotein |  |  |  |  |  |  | 2 |  | 3 |  |  |  |
|  | Metabolic markers |  |  |  |  |  |  | 2 | 4 |  |  |  |  |
|  | CBC |  |  |  |  |  |  | 2 | 4 |  |  |  |  |
|  | Blood chemistry |  |  |  |  |  |  | 2 | 4 |  |  |  |  |
|  | Coagulation profile |  |  |  |  |  |  | 2 | 4 | 6 |  |  |  |
|  | Creatinine clearance |  |  |  |  |  |  | 2 | 4 |  |  |  |  |
|  | HBs AG-6 |  |  |  |  |  |  |  |  | 4 |  |  |  |
|  | HCV antibody-6 |  |  |  |  |  |  |  |  | 1 |  |  |  |
|  | CT/MRI scan |  |  |  |  |  |  |  |  | 1.5 |  |  |  |
| Contact with HCP | GP Appointment |  |  | 2 | 2 | 2 | 2 | 2 | 4 |  |  |  |  |
|  | Dietician Appointment |  |  | 1 | 1 | 1 | 1 | 1 | 1 |  |  |  |  |
|  | Consultant Outpatient Visit |  |  |  |  |  | 1 |  |  | 1.8 |  |  |  |
|  | Outpatient Visits |  |  |  |  |  |  | 2 |  | 5.5 |  |  |  |
|  | Nurse Telephone Consultations |  |  |  |  |  |  |  |  |  |  |  |  |
|  | Hospital Admission - Follow-up |  |  |  |  |  |  |  |  | 30 |  |  |  |
|  | Day Case Follow-up |  |  |  |  |  |  |  |  |  |  |  |  |
|  | Outpatient Visits** |  |  |  |  |  |  | 1 | 5.5 |  |  |  |  |
| Admissions | Emergency Admission - Ascites |  |  |  |  |  |  |  | 2 |  |  |  |  |
|  | Emergency Admission - Variceal Bleed |  |  |  |  |  |  |  | 1.5 |  |  |  |  |
|  | Emergency Admission - Encephalopathy |  |  |  |  |  |  |  | 2 |  |  |  |  |
|  | Planned Admission - Ascites |  |  |  |  |  |  |  | 0.5 |  |  |  |  |
|  | Planned Admission - Variceal Bleeds |  |  |  |  |  |  |  | 0.5 |  |  |  |  |
|  | Hospital Admission - Tumour recurrence |  |  |  |  |  |  |  |  | 1 |  |  |  |
| Procedures | TIPS Stent |  |  |  |  |  |  |  | 0.02 |  |  |  |  |
|  | Day Case, Ascites |  |  |  |  |  |  |  |  |  |  |  |  |
|  | Surgical re-section |  |  |  |  |  |  |  | 1.2 |  |  |  |  |
|  | Radiofrequency ablation (RFA) |  |  |  |  |  |  |  |  |  |  |  |  |
|  | TransArterial chemoembolization (TACE) |  |  |  |  |  |  |  |  |  |  |  |  |
|  | TransArterial radioembolization (TARE) |  |  |  |  |  |  |  |  |  |  |  |  |
|  | Paracentesis |  |  |  |  |  |  |  |  |  |  |  |  |
| Transplant | Liver Transplant |  |  |  |  |  |  |  |  |  |  |  |  |
|  | Liver Re-transplantation |  |  |  |  |  |  |  |  |  |  |  | 0.5 |
|  | Subsequent Care |  |  |  |  |  |  |  |  |  |  |  | 0.95 |
| HCC, hepatocellular carcinoma; HCP, Health care professional; HCRU, Healthcare Resource Use; OGD, Oesophago-gastroduodenoscopy; SoC, Standard of Care; TIPS, Transjugular intrahepatic portosystemic shunt. UAE, United Arab Emirates  **Note**: * The health state LF/LT encompasses three populations: those patients with progressive disease who transition to either “LF/LT with DC (year 1)”, “LF/LT with HCC (year 1)” or “LF/LT (year 2+)”, i.e. Fail DC, Fail HCC and Fail Y2+, respectively. **For Ascites, Variceal Bleeds or Encephalopathy. | | | | | | | | | | | | | |

Supplementary Table 5. Annual HCRU frequencies per Health State for NASH patients on SoC in Kuwait (year 1)

| HCRU | | Health States | | | | | | | | | | | |
| --- | --- | --- | --- | --- | --- | --- | --- | --- | --- | --- | --- | --- | --- |
| Category | Resource | NNAFLD | NAFLD | F0 | F1 | F2 | F3 | CC | DC | HCC | Fail DC* | Fail HCC* | Fail Y2+* |
| Drug | Vitamin E |  |  | 694 | 694 | 694 | 694 |  |  |  |  |  |  |
|  | Pioglitazone |  |  |  |  |  | 183 | 183 |  |  |  |  |  |
|  | Acamprosate |  |  |  |  |  |  |  |  |  |  |  |  |
|  | Obeticholic Acid |  |  |  |  |  |  |  |  |  |  |  |  |
|  | Carvedilol |  |  |  |  |  |  |  |  |  |  |  |  |
|  | Multivitamins |  |  |  |  |  |  | 365 |  |  |  |  |  |
|  | Thiamine |  |  |  |  |  |  |  | 365 |  |  |  |  |
|  | Spironolactone |  |  |  |  |  |  |  | 365 | 73 |  |  |  |
|  | Furosemide |  |  |  |  |  |  |  | 365 | 73 |  |  |  |
|  | Rifaximin |  |  |  |  |  |  |  | 731 |  |  |  |  |
|  | Lactulose |  |  |  |  |  |  |  | 584.4 | 58.4 |  |  |  |
|  | Ceftriaxone |  |  |  |  |  |  |  | 7.5 |  |  |  |  |
|  | Octreotide |  |  |  |  |  |  |  | 6.8 |  |  |  |  |
|  | Normal Saline |  |  |  |  |  |  |  | 32.6 |  |  |  |  |
|  | Terlipressin |  |  |  |  |  |  |  | 2.6 |  |  |  |  |
|  | IV Albumin |  |  |  |  |  |  |  | 3.5 |  |  |  |  |
|  | Midodrine |  |  |  |  |  |  |  | 1.8 |  |  |  |  |
|  | Mycophenolate mofetil |  |  |  |  |  |  |  |  |  | 365 | 365 |  |
|  | Tacrolimus |  |  |  |  |  |  |  |  |  | 2629 | 2629 |  |
|  | Cyclosporine |  |  |  |  |  |  |  |  |  | 7305 | 7305 |  |
|  | Prednisolone |  |  |  |  |  |  |  |  |  | 1836 | 1836 |  |
|  | Valgan |  |  |  |  |  |  |  |  |  | 30 | 30 |  |
|  | Sorafenib |  |  |  |  |  |  |  |  | 95 |  |  |  |
|  | Nivolumab |  |  |  |  |  |  |  |  | 0.11 |  |  |  |
|  | New Treatment |  |  |  |  |  | 365 | 365 |  |  |  |  |  |
| Tests | Fibroscan |  |  | 1 | 1 | 1 | 1 | 1 |  |  |  |  |  |
|  | Liver Function Test |  |  | 2 | 2 | 2 | 2 | 2 | 4 | 4 |  |  |  |
|  | Hep B/C serology |  |  | 1 | 1 | 1 | 1 | 1 | 4 |  |  |  |  |
|  | Ultrasound |  |  | 0.5 | 0.5 | 0.5 | 0.5 | 1 | 2 |  |  |  |  |
|  | Liver Biopsy |  |  |  |  |  |  |  |  | 1 |  |  |  |
|  | Autoimmune Liver Screen |  |  | 1 | 1 | 1 | 1 | 1 | 4 |  |  |  |  |
|  | OGD |  |  |  |  |  |  | 1 | 5 |  |  |  |  |
|  | Ultrasound for HCC |  |  |  |  |  |  | 2 | 4 |  |  |  |  |
|  | Alpha Fetoprotein |  |  |  |  |  |  | 2 |  | 2 |  |  |  |
|  | Metabolic markers |  |  | 2 | 2 | 2 | 2 | 1 | 4 |  |  |  |  |
|  | CBC |  |  | 2 | 2 | 2 | 2 | 1 | 4 | 4 |  |  |  |
|  | Blood chemistry |  |  | 2 | 2 | 2 | 2 | 1 | 4 |  |  |  |  |
|  | Coagulation profile |  |  | 2 | 2 | 2 | 2 | 1 | 4 | 4 |  |  |  |
|  | Creatinine clearance |  |  | 2 | 2 | 2 | 2 | 1 | 4 |  |  |  |  |
|  | HBs AG-6 |  |  |  |  |  |  |  |  | 1 |  |  |  |
|  | HCV antibody-6 |  |  |  |  |  |  |  |  | 1 |  |  |  |
|  | CT/MRI scan |  |  |  |  |  |  |  |  | 2.68 |  |  |  |
| Contact with HCP | GP Appointment |  |  | 2 | 2 | 2 | 2 | 4 | 4 |  |  |  |  |
|  | Dietician Appointment |  |  | 1 | 1 | 1 | 1 |  |  |  |  |  |  |
|  | Consultant Outpatient Visit |  |  |  |  |  |  |  |  | 3 |  |  |  |
|  | Outpatient Visits |  |  |  |  |  |  |  |  | 5.5 |  |  |  |
|  | Nurse Telephone Consultations |  |  |  |  |  |  |  |  |  |  |  |  |
|  | Hospital Admission - Follow-up |  |  |  |  |  |  |  |  |  |  |  |  |
|  | Day Case Follow-up |  |  |  |  |  |  |  |  |  |  |  |  |
|  | Outpatient Visits** |  |  |  |  |  |  |  | 4 |  |  |  |  |
| Admissions | Emergency Admission - Ascites |  |  |  |  |  |  |  | 4 |  |  |  |  |
|  | Emergency Admission - Variceal Bleed |  |  |  |  |  |  |  | 1.5 |  |  |  |  |
|  | Emergency Admission - Encephalopathy |  |  |  |  |  |  |  | 3.5 |  |  |  |  |
|  | Planned Admission - Ascites |  |  |  |  |  |  |  |  |  |  |  |  |
|  | Planned Admission - Variceal Bleeds |  |  |  |  |  |  |  |  |  |  |  |  |
|  | Hospital Admission - Tumour recurrence |  |  |  |  |  |  |  |  | 0.5 |  |  |  |
| Procedures | TIPS Stent |  |  |  |  |  |  |  | 0.02 |  |  |  |  |
|  | Day Case, Ascites |  |  |  |  |  |  |  | 2.4 |  |  |  |  |
|  | Surgical re-section |  |  |  |  |  |  |  |  |  |  | 0.05 |  |
|  | Radiofrequency ablation (RFA) |  |  |  |  |  |  |  |  | 0.02 |  |  |  |
|  | TransArterial chemoembolization (TACE) |  |  |  |  |  |  |  |  | 0.3 |  |  |  |
|  | TransArterial radioembolization (TARE) |  |  |  |  |  |  |  |  | 0.3 |  |  |  |
|  | Paracentesis |  |  |  |  |  |  |  |  | 0.2 |  |  |  |
| Transplant | Liver Transplant |  |  |  |  |  |  |  |  | 0.3 | 1 | 1 |  |
|  | Liver Re-transplantation |  |  |  |  |  |  |  |  |  |  |  |  |
|  | Subsequent Care |  |  |  |  |  |  |  |  |  | 0.95 | 0.95 |  |
| HCC, hepatocellular carcinoma; HCP, Health care professional; HCRU, Healthcare Resource Use; OGD, Oesophago-gastroduodenoscopy; SoC, Standard of Care; TIPS, Transjugular intrahepatic portosystemic shunt.  **Note**: * The health state LF/LT encompasses three populations: those patients with progressive disease who transition to either “LF/LT with DC (year 1)”, “LF/LT with HCC (year 1)” or “LF/LT (year 2+)”, i.e. Fail DC, Fail HCC and Fail Y2+, respectively. **For Ascites, Variceal Bleeds or Encephalopathy. | | | | | | | | | | | | | |

Supplementary Table 6. Annual HCRU frequencies per Health State for NASH patients on SoC in Kuwait (subsequent years)

| HCRU | | Health States | | | | | | | | | | | |
| --- | --- | --- | --- | --- | --- | --- | --- | --- | --- | --- | --- | --- | --- |
| Category | Resource | NNAFLD | NAFLD | F0 | F1 | F2 | F3 | CC | DC | HCC | Fail DC* | Fail HCC* | Fail Y2+* |
| Drug | Vitamin E |  |  | 694 | 694 | 694 | 694 |  |  |  |  |  |  |
|  | Pioglitazone |  |  |  |  |  | 183 | 183 |  |  |  |  |  |
|  | Acamprosate |  |  |  |  |  |  |  |  |  |  |  |  |
|  | Obeticholic Acid |  |  |  |  |  |  |  |  |  |  |  |  |
|  | Carvedilol |  |  |  |  |  |  |  |  |  |  |  |  |
|  | Multivitamins |  |  |  |  |  |  | 365 |  |  |  |  |  |
|  | Thiamine |  |  |  |  |  |  |  | 365 |  |  |  |  |
|  | Spironolactone |  |  |  |  |  |  |  | 183 |  |  |  |  |
|  | Furosemide |  |  |  |  |  |  |  | 183 |  |  |  |  |
|  | Rifaximin |  |  |  |  |  |  |  | 731 |  |  |  |  |
|  | Lactulose |  |  |  |  |  |  |  | 584 |  |  |  |  |
|  | Ceftriaxone |  |  |  |  |  |  |  | 7.5 |  |  |  |  |
|  | Octreotide |  |  |  |  |  |  |  | 6.8 |  |  |  |  |
|  | Normal Saline |  |  |  |  |  |  |  | 32.6 |  |  |  |  |
|  | Terlipressin |  |  |  |  |  |  |  | 2.6 |  |  |  |  |
|  | IV Albumin |  |  |  |  |  |  |  | 3.5 |  |  |  |  |
|  | Midodrine |  |  |  |  |  |  |  | 1.8 |  |  |  |  |
|  | Mycophenolate mofetil |  |  |  |  |  |  |  |  |  |  |  |  |
|  | Tacrolimus |  |  |  |  |  |  |  |  |  |  |  | 329 |
|  | Cyclosporine |  |  |  |  |  |  |  |  |  |  |  | 37 |
|  | Prednisolone |  |  |  |  |  |  |  |  |  |  |  | 114 |
|  | Valgan |  |  |  |  |  |  |  |  |  |  |  |  |
|  | Sorafenib |  |  |  |  |  |  |  |  |  |  |  |  |
|  | Nivolumab |  |  |  |  |  |  |  |  |  |  |  |  |
|  | New Treatment |  |  |  |  |  | 365 | 365 |  |  |  |  |  |
| Tests | Fibroscan |  |  | 1 | 1 | 1 | 1 | 1 |  |  |  |  |  |
|  | Liver Function Test |  |  | 2 | 2 | 2 | 2 | 2 | 4 | 4 |  |  |  |
|  | Hep B/C serology |  |  |  |  |  |  |  | 4 |  |  |  |  |
|  | Ultrasound |  |  | 0.33 | 0.33 | 0.33 | 0.33 | 1 | 2 |  |  |  |  |
|  | Liver Biopsy |  |  |  |  |  |  |  |  |  |  |  |  |
|  | Autoimmune Liver Screen |  |  |  |  |  |  |  | 4 |  |  |  |  |
|  | OGD |  |  |  |  |  |  | 0.5 | 5 |  |  |  |  |
|  | Ultrasound for HCC |  |  |  |  |  |  | 2 |  |  |  |  |  |
|  | Alpha Fetoprotein |  |  |  |  |  |  | 2 |  | 2 |  |  |  |
|  | Metabolic markers |  |  | 2 | 2 | 2 | 2 | 2 | 4 |  |  |  |  |
|  | CBC |  |  | 2 | 2 | 2 | 2 | 2 | 4 | 4 |  |  |  |
|  | Blood chemistry |  |  | 2 | 2 | 2 | 2 | 2 | 4 |  |  |  |  |
|  | Coagulation profile |  |  | 2 | 2 | 2 | 2 | 2 | 4 | 4 |  |  |  |
|  | Creatinine clearance |  |  | 2 | 2 | 2 | 2 | 2 | 4 |  |  |  |  |
|  | HBs AG-6 |  |  |  |  |  |  |  |  | 1 |  |  |  |
|  | HCV antibody-6 |  |  |  |  |  |  |  |  | 1 |  |  |  |
|  | CT/MRI scan |  |  |  |  |  |  |  |  | 1.5 |  |  |  |
| Contact with Health Professional | GP Appointment |  |  | 2 | 2 | 2 | 2 | 4 | 4 |  |  |  |  |
|  | Dietician Appointment |  |  |  |  |  |  |  |  |  |  |  |  |
|  | Consultant Outpatient Visit |  |  |  |  |  |  |  |  | 1.8 |  |  |  |
|  | Outpatient Visits |  |  |  |  |  |  |  |  | 5.5 |  |  |  |
|  | Nurse Telephone Consultations |  |  |  |  |  |  |  |  |  |  |  |  |
|  | Hospital Admission - Follow-up |  |  |  |  |  |  |  |  | 30 |  |  |  |
|  | Day Case Follow-up |  |  |  |  |  |  |  |  |  |  |  |  |
|  | Outpatient Visits** |  |  |  |  |  |  |  | 4 |  |  |  |  |
| Admissions | Emergency Admission - Ascites |  |  |  |  |  |  |  | 4 |  |  |  |  |
|  | Emergency Admission - Variceal Bleed |  |  |  |  |  |  |  | 1.5 |  |  |  |  |
|  | Emergency Admission - Encephalopathy |  |  |  |  |  |  |  | 3.5 |  |  |  |  |
|  | Planned Admission - Ascites |  |  |  |  |  |  |  |  |  |  |  |  |
|  | Planned Admission - Variceal Bleeds |  |  |  |  |  |  |  |  |  |  |  |  |
|  | Hospital Admission - Tumour recurrence |  |  |  |  |  |  |  |  | 0.5 |  |  |  |
| Procedures | TIPS Stent |  |  |  |  |  |  |  | 0.02 |  |  |  |  |
|  | Day Case, Ascites |  |  |  |  |  |  |  |  |  |  |  |  |
|  | Surgical re-section |  |  |  |  |  |  |  | 2.4 |  |  |  |  |
|  | Radiofrequency ablation (RFA) |  |  |  |  |  |  |  |  |  |  |  |  |
|  | TransArterial chemoembolization (TACE) |  |  |  |  |  |  |  |  |  |  |  |  |
|  | TransArterial radioembolization (TARE) |  |  |  |  |  |  |  |  |  |  |  |  |
|  | Paracentesis |  |  |  |  |  |  |  |  |  |  |  |  |
| Transplant | Liver Transplant |  |  |  |  |  |  |  |  |  |  |  |  |
|  | Liver Re-transplantation |  |  |  |  |  |  |  |  |  |  |  | 0.05 |
|  | Subsequent Care |  |  |  |  |  |  |  |  |  |  |  | 0.95 |
| **Key:** HCC, hepatocellular carcinoma; HCRU, Health Care Resource Use; OGD, Oesophago-gastroduodenoscopy; SoC, Standard of Care; TIPS, Transjugular intrahepatic portosystemic shunt.  **Note**: * The health state LF/LT encompasses three populations: those patients with progressive disease who transition to either “LF/LT with DC (year 1)”, “LF/LT with HCC (year 1)” or “LF/LT (year 2+)”, i.e. Fail DC, Fail HCC and Fail Y2+, respectively. **For Ascites, Variceal Bleeds or Encephalopathy. | | | | | | | | | | | | | |

**II. Model Inputs: Health Care Costs for NASH patients on Standard of Care in KSA, UAE and Kuwait**

The model unit costs per category of HCRU are seen in local currency and in USD (Supplementary Table 7 and Supplementary Table 8, respectively).

Supplementary Table 7. Unit Health Care Costs Per Category of HCRU for NASH Patients on SoC in KSA, UAE and Kuwait (local currency)

| HCRU | | Country Unit Costs in Local Currencies | | |
| --- | --- | --- | --- | --- |
| Category | Resource | KSA  (SAR) | UAE  (AED) | Kuwait  (KWD) |
| Drug | Vitamin E | 0.13 | 1.33 | 0.04 |
|  | Pioglitazone | 0.20 | 4.18 | 0.10 |
|  | Acamprosate | 0.00 | 0.00 | 0.00 |
|  | Obeticholic Acid | 0.00 | 0.00 | 0.00 |
|  | Carvedilol | 1.56 | 2.17 | 0.10 |
|  | Multivitamins | 0.12 | 0.17 | 0.01 |
|  | Thiamine | 0.12 | 0.16 | 0.01 |
|  | Spironolactone | 1.22 | 2.25 | 0.18 |
|  | Furosemide | 0.02 | 0.25 | 0.07 |
|  | Rifaximin | 0.00 | 0.00 | 0.00 |
|  | Lactulose | 6.75 | 0.30 | 1.33 |
|  | Ceftriaxone | 300.00 | 220.00 | 18.00 |
|  | Octreotide | 823.5 | 607044.00 | 72192.00 |
|  | Normal Saline | 0.06 | 7.60 | 1.40 |
|  | Terlipressin | 694.16 | 926.75 | 57.45 |
|  | IV Albumin | 52.00 | 50.00 | 5.30 |
|  | Midodrine | 3.46 | 4.82 | 0.29 |
|  | Mycophenolate mofetil | 12.00 | 13.00 | 2.00 |
|  | Tacrolimus | 9.74 | 23.13 | 0.81 |
|  | Cyclosporine | 0.17 | 0.32 | 0.01 |
|  | Prednisolone | 0.16 | 0.54 | 0.02 |
|  | Valgan | 180.00 | 171.00 | 23.40 |
|  | Sorafenib | 698.40 | 763.20 | 61.60 |
|  | Nivolumab | 25388.58 | 23562.00 | 2101.12 |
|  | New Treatment |  |  |  |
| Tests | Fibroscan | 600.00 | 588.00 | 49.66 |
|  | Liver Function Test | 1200.00 | 108.00 | 99.31 |
|  | Hep B/C serology | 128.00 | 141.00 | 10.00 |
|  | Ultrasound | 600.00 | 3441.00 | 20.00 |
|  | Liver Biopsy | 1600.00 | 1105.00 | 132.41 |
|  | Autoimmune Liver Screen | 274.21 | 382.00 | 22.00 |
|  | OGD | 3465.00 | 3397.00 | 50.00 |
|  | Ultrasound for HCC | 600.00 | 235.00 | 5.00 |
|  | Alpha Fetoprotein | 200.00 | 167.00 | 5.00 |
|  | Metabolic markers | 264.88 | 369.00 | 10.00 |
|  | CBC | 43.79 | 61.00 | 1.50 |
|  | Blood chemistry | 301.49 | 420.00 | 12.00 |
|  | Coagulation profile | 96.91 | 135.00 | 12.00 |
|  | Creatinine clearance | 68.91 | 96.00 | 5.70 |
|  | HBs AG-6 | 200.00 | 278.62 | 20.00 |
|  | HCV antibody-6 | 200.00 | 278.62 | 20.00 |
|  | CT/MRI scan | 1175.07 | 1637.00 | 92.50 |
| Contact with HCPs | GP Appointment | 150.00 | 156.00 | 2.00 |
|  | Dietician Appointment | 150.00 | 156.00 | 15.00 |
|  | Consultant Outpatient Visit | 150.00 | 208.97 | 12.41 |
|  | Outpatient Visits | 150.00 | 377.00 | 10.00 |
|  | Nurse Telephone Consultations | 0.00 | 0.00 | 0.00 |
|  | Hospital Admission - Follow-up | 1500.00 | 2089.66 | 124.14 |
|  | Day Case Follow-up | 480.00 | 461.00 | 10.00 |
|  | Outpatient Visits* | 150.00 | 208.97 | 10.00 |
| Admissions | Emergency Admission - Ascites | 1500.00 | 2089.66 | 100.00 |
|  | Emergency Admission - Variceal Bleed | 1500.00 | 2089.66 | 350.00 |
|  | Emergency Admission - Encephalopathy | 1500.00 | 2089.66 | 200.00 |
|  | Planned Admission - Ascites | 2400.00 | 3343.45 | 198.62 |
|  | Planned Admission - Variceal Bleeds | 2400.00 | 3343.45 | 198.62 |
|  | Hospital Admission - Tumour recurrence | 50000.00 | 69655.17 | 10.00 |
| Procedures | TIPS Stent | 20000.00 | 12127.00 | 1655.17 |
|  | Day Case, Ascites | 3000.00 | 4179.31 | 248.28 |
|  | Surgical re-section | 65000.00 | 90551.72 | 5379.31 |
|  | Radiofrequency ablation (RFA) | 30000.00 | 41793.10 | 2482.76 |
|  | TransArterial chemoembolization (TACE) | 75000.00 | 104482.76 | 6206.90 |
|  | TransArterial radioembolization (TARE) | 20000.00 | 278620.69 | 16551.72 |
|  | Paracentesis | 1600.00 | 2228.97 | 132.41 |
| Transplant | Liver Transplant | 902500.00 | 1285550.00 | 99400.00 |
|  | Liver Re-transplantation | 902500.00 | 1285550.00 | 99400.00 |
|  | Subsequent Care | 10000.00 | 13931.03 | 827.59 |
| HCC, hepatocellular carcinoma; HCP, Health care professional; HCRU, Health care resource use; OGD, Oesophago-gastroduodenoscopy; TIPS, Transjugular intrahepatic portosystemic shunt. SAR, Saudi Riyal; SoC, Standard of care; AED, United Arab Emirates Dirham; KWD, Kuwaiti Dinar  **Note**: * For Ascites, Variceal Bleeds or Encephalopathy. | | | | |

Supplementary Table 8. Unit Health Care Costs Per Category of HCRU for NASH Patients on SoC in KSA, UAE and Kuwait (USD 2018)

| HCRU | | Country Unit Costs Converted to USD (2018) | | |
| --- | --- | --- | --- | --- |
| Category | Resource | KSA | UAE | Kuwait |
| Drug | Vitamin E | 0.04 | 0.36 | 0.14 |
|  | Pioglitazone | 0.05 | 1.13 | 0.32 |
|  | Acamprosate | 0.00 | 0.00 | 0.00 |
|  | Obeticholic Acid | 0.00 | 0.00 | 0.00 |
|  | Carvedilol | 0.42 | 0.59 | 0.35 |
|  | Multivitamins | 0.03 | 0.05 | 0.03 |
|  | Thiamine | 0.03 | 0.04 | 0.03 |
|  | Spironolactone | 0.33 | 0.61 | 0.59 |
|  | Furosemide | 0.01 | 0.07 | 0.24 |
|  | Rifaximin | 0.00 | 0.00 | 0.00 |
|  | Lactulose | 1.82 | 0.08 | 4.39 |
|  | Ceftriaxone | 81.00 | 59.40 | 59.40 |
|  | Octreotide | 222.34 | 264.64 | 384.65 |
|  | Normal Saline | 0.02 | 2.05 | 4.62 |
|  | Terlipressin | 187.42 | 250.22 | 189.58 |
|  | IV Albumin | 14.04 | 13.50 | 17.49 |
|  | Midodrine | 0.93 | 1.30 | 0.94 |
|  | Mycophenolate mofetil | 3.24 | 3.51 | 6.60 |
|  | Tacrolimus | 2.63 | 6.24 | 2.66 |
|  | Cyclosporine | 0.05 | 0.09 | 0.04 |
|  | Prednisolone | 0.04 | 0.15 | 0.05 |
|  | Valgan | 48.60 | 46.17 | 77.22 |
|  | Sorafenib | 188.57 | 206.06 | 203.28 |
|  | Nivolumab | 6854.92 | 6361.74 | 6933.71 |
|  | New Treatment |  |  |  |
| Tests | Fibroscan | 162.00 | 158.76 | 163.86 |
|  | Liver Function Test | 324.00 | 29.16 | 327.72 |
|  | Hep B/C serology | 34.56 | 38.07 | 33.00 |
|  | Ultrasound | 162.00 | 929.07 | 66.00 |
|  | Liver Biopsy | 432.00 | 298.35 | 436.97 |
|  | Autoimmune Liver Screen | 74.04 | 103.14 | 72.60 |
|  | OGD | 935.55 | 917.19 | 165.00 |
|  | Ultrasound for HCC | 162.00 | 63.45 | 16.50 |
|  | Alpha Fetoprotein | 54.00 | 45.09 | 16.50 |
|  | Metabolic markers | 71.52 | 99.63 | 33.00 |
|  | CBC | 11.82 | 16.47 | 4.95 |
|  | Blood chemistry | 81.40 | 113.40 | 39.60 |
|  | Coagulation profile | 26.16 | 36.45 | 39.60 |
|  | Creatinine clearance | 18.61 | 25.92 | 18.82 |
|  | HBs AG-6 | 54.00 | 75.23 | 66.00 |
|  | HCV antibody-6 | 54.00 | 75.23 | 66.00 |
|  | CT/MRI scan | 317.27 | 441.99 | 305.25 |
| Contact with HCPs | GP Appointment | 40.50 | 42.12 | 6.60 |
|  | Dietician Appointment | 40.50 | 42.12 | 49.50 |
|  | Consultant Outpatient Visit | 40.50 | 56.42 | 40.97 |
|  | Outpatient Visits | 40.50 | 101.79 | 33.00 |
|  | Nurse Telephone Consultations | 0.00 | 0.00 | 0.00 |
|  | Hospital Admission - Follow-up | 405.00 | 564.21 | 409.66 |
|  | Day Case Follow-up | 129.60 | 124.47 | 33.00 |
|  | Outpatient Visits* | 40.50 | 56.42 | 33.00 |
| Admissions | Emergency Admission - Ascites | 405.00 | 564.21 | 330.00 |
|  | Emergency Admission - Variceal Bleed | 405.00 | 564.21 | 1155.00 |
|  | Emergency Admission - Encephalopathy | 405.00 | 564.21 | 660.00 |
|  | Planned Admission - Ascites | 648.00 | 902.73 | 655.45 |
|  | Planned Admission - Variceal Bleeds | 648.00 | 902.73 | 655.45 |
|  | Hospital Admission - Tumour recurrence | 13500.00 | 18806.90 | 33.00 |
| Procedures | TIPS Stent | 5400.00 | 3274.29 | 5462.07 |
|  | Day Case, Ascites | 810.00 | 1128.41 | 819.31 |
|  | Surgical re-section | 17550.00 | 24448.97 | 17751.72 |
|  | Radiofrequency ablation (RFA) | 8100.00 | 11284.14 | 8193.10 |
|  | TransArterial chemoembolization (TACE) | 20250.00 | 28210.34 | 20482.76 |
|  | TransArterial radioembolization (TARE) | 54000.00 | 75227.59 | 54620.69 |
|  | Paracentesis | 432.00 | 601.82 | 436.97 |
| Transplant | Liver Transplant | 243675.00 | 347098.50 | 328020.00 |
|  | Liver Re-transplantation | 243675.00 | 347098.50 | 328020.00 |
|  | Subsequent Care | 2700.00 | 3761.38 | 2731.03 |
| HCC, hepatocellular carcinoma; HCP, Health Care Professional; HCRU, Health Care Resource Ues; OGD, Oesophago-gastroduodenoscopy; SoC, Standard of Care; TIPS, Transjugular intrahepatic portosystemic shunt; USD, US dollar.  **Note**: * For Ascites, Variceal Bleeds or Encephalopathy. | | | | |

**III. Model Inputs: Health State Utility Values for NASH patients on Standard of Care in KSA, UAE and Kuwait.**

In the absence of Middle East (ME) country-specific utility values for model health-states, UK utility values^(^^[[2]](#endnote-2))^ informed the health-states, except for the health-states for non-NAFLD and NAFLD (as both were reported as 1.00)^(2)^. Given that the UK population norms for all age groups and genders are <1, the UK population norm (utility 0.88) was assumed to apply for both the non-NAFLD and NAFLD health-state^(^^[[3]](#endnote-3),^^[[4]](#endnote-4),^^[[5]](#endnote-5))^. Utilities used in the modelling in all three ME countries are seen in Supplementary Table 9.

Supplementary Table 9. Health State Utility Values (KSA, UAE & Kuwait)

| Health State | Mean Utility | LB | UB |
| --- | --- | --- | --- |
| Non-NAFLD | 0.88 | 0.86 | 0.90 |
| NAFLD | 0.88 | 0.86 | 0.90 |
| F0 | 0.85 | 0.84 | 0.86 |
| F1 | 0.84 | 0.83 | 0.85 |
| F2 | 0.84 | 0.83 | 0.85 |
| F3 | 0.84 | 0.83 | 0.85 |
| CC | 0.80 | 0.65 | 0.89 |
| DC | 0.60 | 0.49 | 0.81 |
| HCC | 0.73 | 0.50 | 0.80 |
| *LF/LT with DC or HCC (year 1) - Fail Y1 | 0.69 | 0.62 | 0.86 |
| *LF/LT (year 2+) - Fail Y2+ | 0.80 | 0.79 | 0.83 |
| Dead | 0.00 | 0 | 0 |
| CC, compensated cirrhosis; DC, decompensated cirrhosis; Fail Y1, first year following liver failure; Fail Y2+, subsequent years following liver failure; F0-F3, fibrosis score 0-3; HCC, hepatocellular carcinoma; LB, lower bound; NAFLD, non-alcoholic fatty liver disease; NASH, non- alcoholic steatohepatitis; NAFLD,NAFLD population; UB, upper bound.  Note: *The health state LF/LT encompasses three populations: those patients with progressive disease who transition to either “LF/LT with DC (year 1)”, “LF/LT with HCC (year 1)” or “LF/LT (year 2+)” | | | |

Lower and upper bounds were assumed to be ±5% of the mean utility.

**REFERENCES**

1. Digitize graphs and plots - GetData Graph Digitizer - graph digitizing software [Internet]. [cited 2018 Dec 20]. Available from: http://getdata-graph-digitizer.com/ [↑](#endnote-ref-1)
2. Zhang E, Wartelle-Bladou C, Lepanto L, Lachaine J, Cloutier G, Tang A. Cost-utility analysis of nonalcoholic steatohepatitis screening. European Radiology 2015;25(11):3282-3294. [↑](#endnote-ref-2)
3. Alswat K, Aljumah AA, Sanai FM, Abaalkhail F, Alghamdi M, Al-Hamoudi WK, et al. Nonalcoholic fatty liver disease burden – Saudi Arabia and United Arab Emirates, 2017–2030. Saudi J Gastroenterol 2018;24(4):211-19 [↑](#endnote-ref-3)
4. Al-Hamoudi, W, El-Sabbah, M, Ali, S, Altuwaijri, M, Bedewi, M, Mustafa, A et al. Epidemiological, clinical, and biochemical characteristics of Saudi patients with nonalcoholic fatty liver disease: A hospital-based study. Annals of Saudi Medicine 2012; 32: 288-92. [↑](#endnote-ref-4)
5. Ara R, Brazier JE. Using Health State Utility Values from the General Population to Approximate Baselines in Decision Analytic Models when Condition-Specific Data are Not Available. Value in Health 2011;14(4):539-545. [↑](#endnote-ref-5)
