# Supplementary material for: Clinical and economic burden of nonalcoholic steatohepatitis in Saudi Arabia, United Arab Emirates and Kuwait
Source: Hepatol Int. 2021 Apr 6;15(4):912–21. doi: 10.1007/s12072-021-10182-x (PMC8382637; doi:10.1007/s12072-021-10182-x)
Supplement: Supplementary file 2 — Supplementary file2 (DOCX 520 kb) [file 12072_2021_10182_MOESM2_ESM.docx]

**ARTICLE TITLE: Clinical and economic burden of nonalcoholic steatohepatitis in Saudi Arabia, United Arab Emirates and Kuwait**

**JOURNAL NAME**: Hepatology International

**AUTHOR NAMES**: Faisal M Sanai, Abdullah Al Khathlan, Ahmad Al Fadhli, Ahmad S Jazzar, Al Moutaz Hashim, Eid Mansour, Faisal Abaalkhail, Fuad Hasan, Hajer Al Mudaiheem, Huda Al Quraishi, Juliana Bottomley, Khalid A Alswat, Mohammed Al Ghamdi, Mohamed Farghaly, Motaz Fathy, Nancy Awad, Omneya Mohamed, Sam Kozma, Waleed Al-Hamoudi and Ahmed Al-jedai

**CORRESPONDING AUTHOR**: Professor Ahmed Al-jedai, Therapeutic Affairs Deputyship, Ministry of Health, Saudi Arabia. E-mail: [ahaljedai@moh.gov.sa](mailto:ahaljedai@moh.gov.sa)

**SUPPLEMENTARY MATERIAL 2.**  **Model Results**.

1. New cases in UAE and Kuwait
2. Lifetime QALYs and QALYs per model health state on Standard of Care in KSA, UAE and Kuwait
3. Total Costs, and Breakdown of Costs by Health State Incurred and Cost Category of Standard of Care in KSA, UAE and Kuwait from 2018 to 2030 (in local currencies)

**1. New Cases of NASH in UAE and Kuwait**

Similar trends in escalating new cases to those predicted in KSA (main manuscript) were also estimated in UAE and Kuwait (Supplementary Figures 1 and 2, respectively). In UAE cases per year with F0-F3 increased by 79% from 43,004 to 77,137 (Supplementary Figure 1A), patients with CC, DC and HCC rose from 2,275 to 6,586 (189% increase) (Supplementary Figure 1B), those with LF/LT increased 338% (from 29 in 2018 to 127 in 2030) (Supplementary Figure 1C) while there were 6085 LD from 2018-2030 (Supplementary Figure 1D).

Supplementary Figure 1. New cases annually per model health-state in UAE (local population). NASH patients on Standard of Care (2018-2030): A) fibrosis stages F0, F1, F2 and F3; B) compensated cirrhosis, decompensated cirrhosis and hepatocellular carcinoma; C) liver failure or liver transplant and D) liver-related death

Supplementary Figure 2A describes a similar increase of F0-F3 cases annually from 174,397 to 310,149 by 2030 in Kuwait, 189% increase in new cases with CC, DC and HCC (9,225 to 26,652; Supplementary Figure 2B), >three-fold increase in cases of LF/LT (117 patients in 2018 to 514 in 2030; Supplementary Figure 2C) while 24,646 LD patients were estimated from 2018-2030 in Kuwait (Supplementary Figure 2D).

Supplementary Figure 2. New cases annually per model health-state in Kuwait. NASH patients on Standard of Care (2018-2030): A) fibrosis stages F0, F1, F2 and F3; B) compensated cirrhosis, decompensated cirrhosis and hepatocellular carcinoma; C) liver failure or liver transplant and D) liver-related death

**2. Lifetime QALYs and QALYs per model health state on Standard of Care in KSA, UAE and Kuwait**

Supplementary Table 1 describes the total QALYs and life years (LYs) predicted per country adopting current SoC management patterns in NASH.

Supplementary Table 1. QALYs and LYs (thousands) from 2018-2030: NASH patients on Standard of Care in KSA, UAE and Kuwait.

| Health-State | KSA | | UAE | | Kuwait | |
| --- | --- | --- | --- | --- | --- | --- |
|  | **QALYs** | **LYs** | **QALYs** | **LYs** | **QALYs** | **LYs** |
| F0 | 4,200 | 4,900 | 130.1 | 153.0 | 522.8 | 615.1 |
| F1 | 7,300 | 8,600 | 225.6 | 268.4 | 911.8 | 1,100 |
| F2 | 3,800 | 4,500 | 117.0 | 139.2 | 473.6 | 563.8 |
| F3 | 2,100 | 2,500 | 66.1 | 78.7 | 267.6 | 318.6 |
| CC | 718.1 | 897.7 | 22.3 | 27.8 | 90.3 | 112.8 |
| DC | 125.2 | 208.7 | 3.9 | 6.5 | 15.7 | 26.2 |
| HCC | 261.9 | 358.7 | 8.1 | 11.1 | 32.9 | 45.1 |
| LF/LT with DC (year 1) - Fail DC | 2.9 | 4.1 | 0.09 | 0.13 | 0.36 | 0.52 |
| LF/LT with HCC (year 1) - Fail HCC | 4.6 | 6.7 | 0.14 | 0.21 | 0.58 | 0.85 |
| LF/LT (year 2+) - Fail Y2+ | 11.6 | 14.6 | 0.36 | 0.45 | 1.47 | 1.83 |
| **TOTAL** | **18,524** | **21,990** | **573** | **685** | **2,317** | **2,784** |
| Note: Health-state LF/LT encompasses three populations: patients with progressive disease transitioning to “LF/LT with DC (year 1)”, “LF/LT with HCC (year 1)” or “LF/LT (year 2+)”.  Abbreviations: CC, compensated cirrhosis; DC, decompensated cirrhosis; Fail DC, liver failure with decompensated cirrhosis; Fail HCC, liver failure with hepatocellular carcinoma; Fail Y2+, liver failure for 2 or more years; F0-F3, fibrosis score 0-3; HCC, hepatocellular carcinoma; KSA, Kingdom of Saudi Arabia; LY, life year; NASH, nonalcoholic steatohepatitis; QALY, quality adjusted life year; UAE, United Arab Emirates | | | | | | |

Due to KSA, UAE and Kuwait adopting the same transition probabilities for SoC management in the model, the proportions of QALYs accrued in each health-state are equal in all countries (Supplementary Figure 3). Thus, the model predicts that the majority of QALYs (94%) accrued while in initial fibrosis stages of NASH (F0-F3). It is also clear that with disease progression (CC, DC or HCC), very few QALYs are accrued

Supplementary Figure 3. QALYs per model health-state. NASH patients on Standard of Care in KSA, UAE and Kuwait

**3.Total Costs, and Breakdown of Costs by Health State Incurred and Cost Category of Standard of Care in KSA, UAE and Kuwait from 2018 to 2030 (in local currencies)**

Based upon expert estimated HCRU incurred per health state (Online Resource 1, Supplementary Tables 1 to 6) and corresponding unit cost per category of HCRU in each country (Online Resource 1, Supplementary Table 7), the annual health state costs for SoC in model were estimated (in local currency), as seen in Supplementary Table 2 below.

Supplementary Table 2. Annual Health Care Costs Per Health State for NASH patients on SoC in KSA, UAE and Kuwait in local currencies

| Country | Year | Health States | | | | | | | |
| --- | --- | --- | --- | --- | --- | --- | --- | --- | --- |
|  |  | F0 | F1 | F2 | F3 | CC | DC | HCC | LF/LT* |
| **KSA**  (SAR) | 1 | 5,119 | 5,119 | 5,919 | 6,105 | 11,510 | 68,717 | 443,604 | 1,900,549 |
|  | 2+ | 3,191 | 3,191 | 3,991 | 4,027 | 8,213 | 50,717 | 81,894 | 63,868 |
| **UAE**  (AED) | 1 | 7,046 | 7,046 | 7,830 | 12,199 | 19,647 | 54,395 | 629,408 | 2,748,384 |
|  | 2+ | 1,785 | 1,785 | 2,569 | 3,541 | 6,026 | 164,620 | 140,602 | 85,188 |
| **Kuwait**  (KWD) | 1 | 420 | 420 | 420 | 438 | 441 | 5,257 | 41,488 | 207,925 |
|  | 2+ | 370 | 370 | 370 | 387 | 425 | 17,552 | 4,446 | 6,023 |
| F0-F3, fibrosis score 0 to 3; CC, compensated cirrhosis; DC, decompensated cirrhosis; HCC, hepatocellular carcinoma; LF, liver failure; LT, liver transplant. SAR, Saudi Riyal; AED, United Arab Emirates Dirham; KWD, Kuwaiti Dinar  *Note: The health state LF/LT encompasses three populations: those patients with progressive disease who transition to either “LF/LT with DC (year 1)”, “LF/LT with HCC (year 1)” or “LF/LT (year 2+)”, i.e. Fail DC, Fail HCC and Fail Y2+, respectively | | | | | | | | | |

The total lifetime national discounted NASH costs in KSA, UAE and Kuwait, assuming no change in SoC, are seen in local currencies (Supplementary Table 3) overleaf.

Supplementary Table 3. Total Discounted Lifetime Costs of SoC in KSA, UAE and Kuwait from 2018 to 2030 (in local currencies)

| Health State | KSA Costs  (SAR) | UAE Costs  (AED) | Kuwait Costs  (KWD) |
| --- | --- | --- | --- |
| NASH F0 | 18,593.3 m | 525.2 m | 237.2 m |
| NASH F1 | 29,530.1 m | 646.3 m | 407.9 m |
| NASH F2 | 18,840.7 m | 437.4 m | 211.6 m |
| NASH F3 | 11,839.7 m | 490.0 m | 128.4 m |
| CC | 8,942.4 m | 369.1 m | 48.9 m |
| DC | 11,190.2 m | 950.0 m | 408.4 m |
| HCC | 39,765.7 m | 2000.2 m | 334.3 m |
| *LF/LT with DC (year 1) - Fail DC | 3,944.3 m | 176.9 m | 54.3 m |
| *LF/LT with HCC (year 1) - Fail HCC | 6,409.4 m | 287.5 m | 88.1 m |
| *LF/LT (year 2+) - Fail Y2+ | 628.3 m | 26.0 m | 7.4 m |
| **TOTAL** | **149.68 bn** | **5.91 bn** | **1.93 bn** |
| **Key:** CC, compensated cirrhosis; DC, decompensated cirrhosis; Fail DC, liver failure with decompensated cirrhosis; Fail HCC, liver failure with hepatocellular carcinoma; Fail Y2+, liver failure for 2 or more years; F0-F3, fibrosis score 0-3; HCC, hepatocellular carcinoma; k, thousand(s); LY, life year; m, million(s); NASH, non- alcoholic steatohepatitis; SAR, Saudi Riyal; AED, United Arab Emirates Dirham; KWD, Kuwaiti Dinar  *Note: The health state LF/LT encompasses three populations: those patients with progressive disease who transition to either “LF/LT with DC (year 1)”, “LF/LT with HCC (year 1)” or “LF/LT (year 2+)”, i.e. Fail DC, Fail HCC and Fail Y2+, respectively | | | |

The predicted lifetime discounted costs in local currencies listed in Supplementary Table 3 are also displayed pictorially a) according to the health state in which they occurred and b) according to cost category in the following Supplementary Figures 4, 5 and 6 for KSA, UAE and Kuwait, respectively.

Supplementary Figure 4. KSA – SoC (SAR)

a). Breakdown by health state in which they incurred in KSA


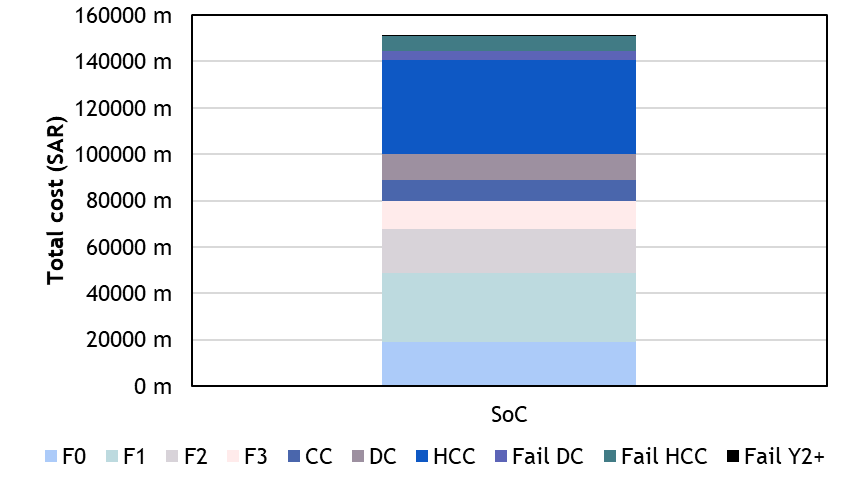


b). Breakdown of costs by cost category in KSA


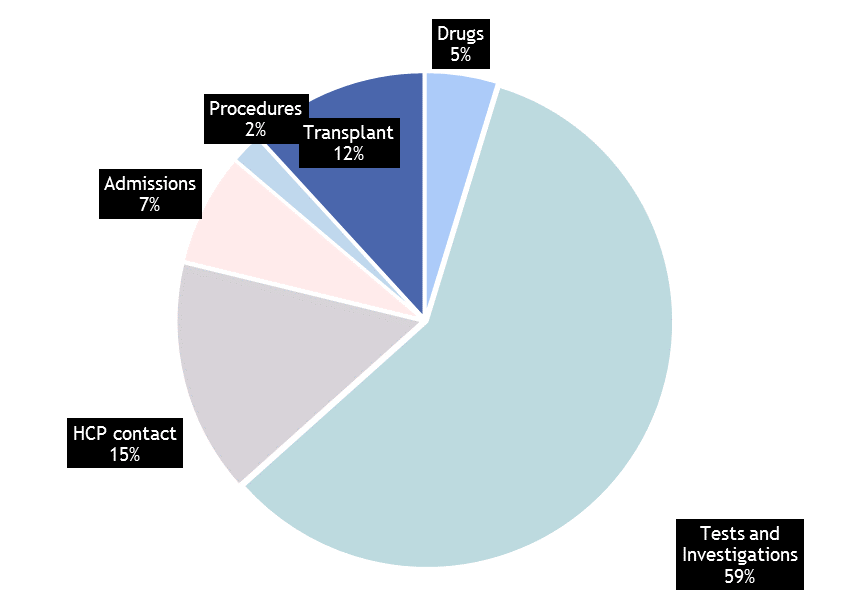


CC, compensated cirrhosis; DC, decompensated cirrhosis; Fail DC, liver failure with decompensated cirrhosis; Fail HCC, liver failure with hepatocellular carcinoma; Fail Y2+, liver failure for 2 or more years; F0-F3, fibrosis score 0-3; HCC, hepatocellular carcinoma; KSA, Kingdom of Saudi Arabia; SoC, standard of care; HCP, Healthcare professional; SAR, Saudi Riyal

Supplementary Figure 5 UAE – SoC (AED)

a). Breakdown by health state in which they incurred in UAE


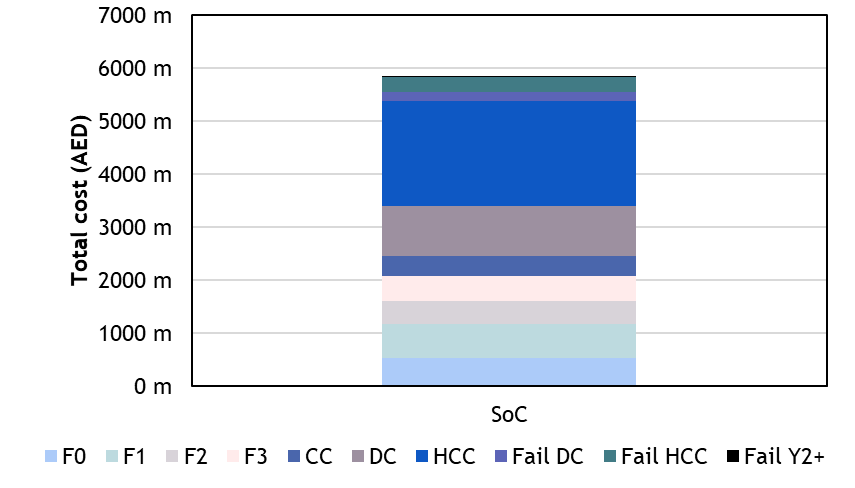


b). Breakdown of costs by cost category in UAE


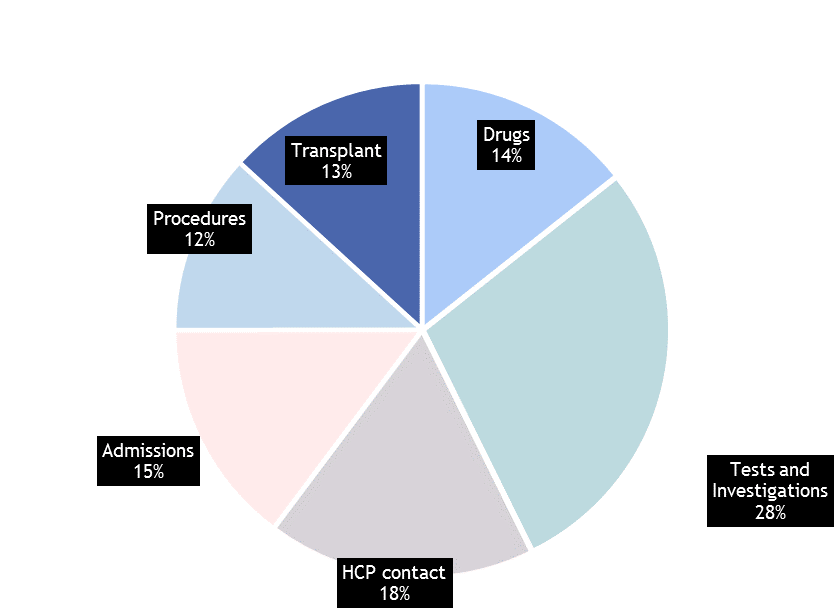


**Note**: The health state LF/LT encompasses three populations: those patients with progressive disease who transition to either “LF/LT with DC (year 1)”, “LF/LT with HCC (year 1)” or “LF/LT (year 2+)”, i.e. Fail DC, Fail HCC and Fail Y2+, respectively

**Key:** AED, United Arab Emirates Dirham; CC, compensated cirrhosis; DC, decompensated cirrhosis; Fail DC, liver failure with decompensated cirrhosis; Fail HCC, liver failure with hepatocellular carcinoma; Fail Y2+, liver failure for 2 or more years; F0-F3, fibrosis score 0-3; HCC, hepatocellular carcinoma; SoC, standard of care; HCP, health care professional; UAE, United Arab Emirates

Supplementary Figure .6. Kuwait – SoC (KWD)

a). Breakdown by health state in which they incurred in Kuwait


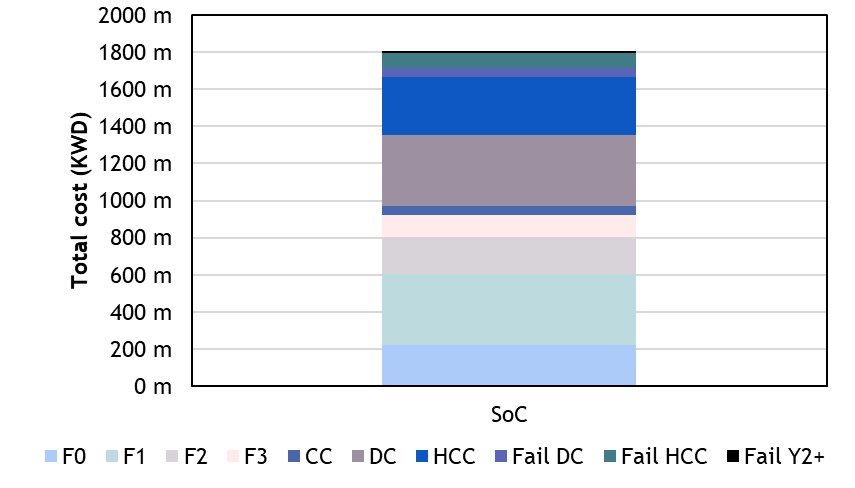


b). Breakdown of costs by cost category in Kuwait


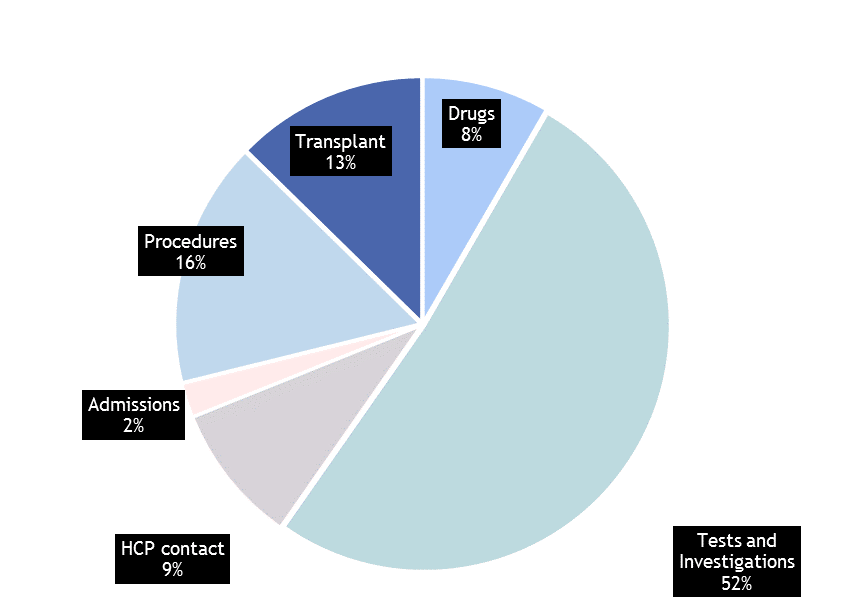


**Note**: The health state LF/LT encompasses three populations: those patients with progressive disease who transition to either “LF/LT with DC (year 1)”, “LF/LT with HCC (year 1)” or “LF/LT (year 2+)”, i.e. Fail DC, Fail HCC and Fail Y2+, respectively

**Key:** CC, compensated cirrhosis; DC, decompensated cirrhosis; Fail DC, liver failure with decompensated cirrhosis; Fail HCC, liver failure with hepatocellular carcinoma; Fail Y2+, liver failure for 2 or more years; F0-F3, fibrosis score 0-3; HCC, hepatocellular carcinoma; SoC, standard of care; HCP, Health care professional; KWD, Kuwaiti Dinar
