# Supplementary material for: Clinical and economic burden of nonalcoholic steatohepatitis in Saudi Arabia, United Arab Emirates and Kuwait
Source: Hepatol Int. 2021 Apr 6;15(4):912–21. doi: 10.1007/s12072-021-10182-x (PMC8382637; doi:10.1007/s12072-021-10182-x)
Supplement: Supplementary file 3 — Supplementary file3 (DOCX 48 kb) [file 12072_2021_10182_MOESM3_ESM.docx]

**ARTICLE TITLE: Clinical and economic burden of nonalcoholic steatohepatitis in Saudi Arabia, United Arab Emirates and Kuwait**

**JOURNAL NAME**: Hepatology International

**AUTHOR NAMES**: Faisal M Sanai, Abdullah Al Khathlan, Ahmad Al Fadhli, Ahmad S Jazzar, Al Moutaz Hashim, Eid Mansour, Faisal Abaalkhail, Fuad Hasan, Hajer Al Mudaiheem, Huda Al Quraishi, Juliana Bottomley, Khalid A Alswat, Mohammed Al Ghamdi, Mohamed Farghaly, Motaz Fathy, Nancy Awad, Omneya Mohamed, Sam Kozma, Waleed Al-Hamoudi and Ahmed Al-jedai

**CORRESPONDING AUTHOR**: Professor Ahmed Al-jedai, Therapeutic Affairs Deputyship, Ministry of Health, Saudi Arabia. E-mail: [ahaljedai@moh.gov.sa](mailto:ahaljedai@moh.gov.sa)

**SUPPLEMENTARY MATERIAL 3.** Estimated National Health Care Spending Attributable to Standard of Care in NASH

1. KSA, UAE and Kuwait in 2019 [Supplementary Table 1]
2. USA, Germany, France, Italy and UK in 2016 [Supplementary Table 2]

Supplementary Table 1: NASH DIRECT COSTS AS A PERCENTAGE OF HEALTH CARE SPENDING IN KSA, UAE AND KUWAIT

| **KSA** |  | 2018 | **2019** | 2020 | 2021 | 2022 | 2023 | 2024 |
| --- | --- | --- | --- | --- | --- | --- | --- | --- |
|  | Discounted cost (USD million) | 2351.7 | 2483.7 | 2615.9 | 2747.3 | 2877.1 | 3004.7 | 3129.6 |
|  | Health spend (USD million)^[[1]](#footnote-1)^ | 40659 | 42567 | 44309 | 46291 | 48410 | 50663 | 53081 |
|  | **NASH costs as proportion health spend (%)** | **5.78** | **5.83** | **5.90** | **5.93** | **5.94** | **5.93** | **5.90** |
| **UAE** | Discounted cost* (USD million) | 90.2 | 95.4 | 100.7 | 106.2 | 111.7 | 117.3 | 122.9 |
|  | Health spend (USD million)^1^ | 13563 | 14308 | 15124 | 16024 | 17028 | 18142 | 19380 |
|  | Health spend adjusted for Emiratis only (USD, million)* | 1559.7 | 1645.4 | 1739.3 | 1842.8 | 1958.2 | 2086.3 | 2228.7 |
|  | **NASH costs as proportion health spend (%)** | **5.79** | **5.80** | **5.79** | **5.76** | **5.71** | **5.62** | **5.52** |
| **Kuwait** | Discounted cost (USD million) | 395.9 | 408.9 | 423.3 | 438.6 | 454.7 | 471.2 | 488.0 |
|  | Health spend (USD million)^1^ | 5077 | 5338 | 5600 | 5996 | 6427 | 6870 | 7324 |
|  | **NASH costs as proportion health spend (%)** | **7.80** | **7.66** | **7.56** | **7.32** | **7.07** | **6.86** | **6.66** |
| **Key:** *Local Emiratis population in UAE only; CC, compensated cirrhosis; DC, decompensated cirrhosis; Fail DC, liver failure with decompensated cirrhosis; Fail HCC, liver failure with hepatocellular carcinoma; Fail Y2+, liver failure for 2 or more years; F0-F3, fibrosis score 0-3; HCC, hepatocellular carcinoma; KSA, Kingdom of Saudi Arabia; NASH, nonalcoholic steatohepatitis; UAE, United Arab Emirates | | | | | | | | |

Supplementary Table 2: NASH DIRECT COSTS AS A PERCENTAGE OF HEALTH CARE SPENDING IN USA, GERMANY, FRANCE, ITALY AND UK

While recent comparative percentage of health spend attributable to NASH data are not available to the authors, comparative percentage of health spend attributable to NASH available for 2015-2016 was possible to calculate. This was estimated via data presented in Table 5 of Younossi et al (2016)^[[2]](#footnote-2)^ and reference to available population and healthcare spend for the period 2015-2016^[[3]](#footnote-3)^.

| **Variable for 2015-2016** | **USA** | **Germany** | **France** | **Italy** | **UK** | **Source** |
| --- | --- | --- | --- | --- | --- | --- |
| Total annual direct NAFLD costs (USD, billions) | 103.31 | 4.33 | 11.40 | 11.95 | 5.24 | Younossi et al 2016, Table 5, Row 1 |
| Total annual direct NASH costs (USD, billions) in 2016 | 16.75 | 0.84 | 2.24 | 2.17 | 0.91 | Calculation. Table 5 in Younossi et al 2016: Total NAFLD costs (row 1) minus costs due to NAFL (row 7 data) |
| Population (millions) | 321.37 | 80.85 | 66.55 | 61.85 | 64.09 | <https://www.emergobyul.com/resources/worldwide-health-expenditures> |
| Total Healthcare Spend 2015 (USD, billion) | 3,021 | 437 | 330 | 201 | 252 | <https://www.emergobyul.com/resources/worldwide-health-expenditures> |
| **% health spend due to NASH** | **0.55%** | **0.19%** | **0.68%** | **1.08%** | **0.36%** | Calculation. Total direct NASH costs divided by Total health spend (%) |


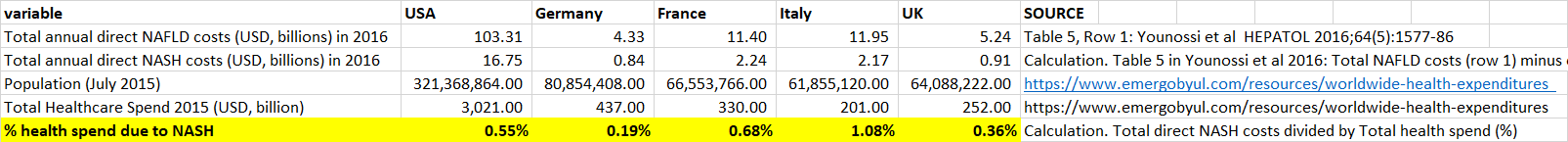


1. Health spend estimates for KSA, UAE and Kuwait available from www.ftchsolutions.com [↑](#footnote-ref-1)
2. Younossi ZM, Blissett D, Blissett R, Henry L, Stepanova M, Younossi Y et al 2016. The economic and clinical burden of nonalcoholic fatty liver disease in the United States and Europe. HEPATOLOGY 2016;64:1577-1586 [↑](#footnote-ref-2)
3. Health Care Spending by Country. Available at: <https://www.emergobyul.com/resources/worldwide-health-expenditures> (Data compiled in July 2016) [↑](#footnote-ref-3)
